# Intramacrophage ROS Primes the Innate Immune System via JAK/STAT and Toll Activation

**DOI:** 10.1016/j.celrep.2020.108368

**Published:** 2020-11-10

**Authors:** Sveta Chakrabarti, Sandhya S. Visweswariah

**Affiliations:** 1 Department of Molecular Reproduction, Development and Genetics, Indian Institute of Science, Bengaluru, India

**Keywords:** *Drosophila*, hemocyte, upd-3, injury, JAK/STAT signaling, Toll pathway, reactive oxygen species, trained immunity

## Abstract

Tissue injury is one of the most severe environmental perturbations for a living organism. When damage occurs in adult *Drosophila*, there is a local response of the injured tissue and a coordinated action across different tissues to help the organism overcome the deleterious effect of an injury. We show a change in the transcriptome of hemocytes at the site of tissue injury, with pronounced activation of the Toll signaling pathway. We find that induction of the cytokine upd-3 and Toll receptor activation occur in response to injury alone, in the absence of a pathogen. Intracellular accumulation of hydrogen peroxide in hemocytes is essential for upd-3 induction and is facilitated by the diffusion of hydrogen peroxide through a channel protein Prip. Importantly, hemocyte activation and production of reactive oxygen species (ROS) at the site of a sterile injury provide protection to flies on subsequent infection, demonstrating training of the innate immune system.

## Highlights

• H_2_O_2_ production from a wound activates hemocytes and increases expression of *upd3*
• A fly aquaporin contributes to increased intracellular ROS in hemocytes following injury
• The Src42A/Shark/Draper pathway regulates *upd3* expression in hemocytes
• The Toll pathway is induced on injury and protects flies from subsequent infection

## Graphical Abstract

**Figure:**
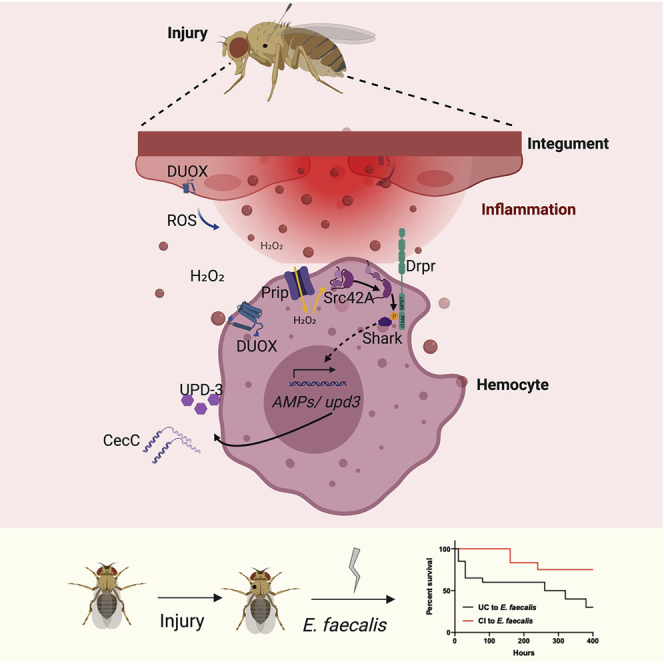

## Teaser

Chakrabarti and Visweswariah show that intra-hemocyte ROS is required to activate Toll signaling and induction of upd3 in hemocytes upon injury. Duox is required in hemocytes to increase H_2_O_2_ levels, which is facilitated by H_2_O_2_ diffusion through a channel protein, Prip. Toll activation protects from subsequent infection with *Enterococcus faecali*.

## Introduction

The adult *Drosophila* is an excellent model to study host responses to infection and injury (Lemaitre and Hoffmann, 2007). The cellular immune arm in *Drosophila* comprises cells called hemocytes. They circulate freely in the body cavity of *Drosophila*, function as professional phagocytes of the immune system, and therefore can be considered the fly equivalent of bone-marrow-derived macrophages. Molecular details of how hemocytes contribute to host development reveal their role in clearing apoptotic corpses and patterning tissues in *Drosophila* (Wood and Martin, 2017). However, once development is complete, very little is known about whether hemocytes play roles that contribute to the overall health of the host. A few studies have shown that *Drosophila* hemocytes maintain host health through their ability to carry out long-range communication to other tissues during a systemic wound response (Lee and Miura, 2014). Hemocytes therefore serve as messengers between the wound site and distant tissues such as the fat body and intestinal tract (Agaisse et al., 2003; Chakrabarti et al., 2016).

In response to physical or chemical injuries, organisms must activate multiple wound-repair systems at the cellular, tissue, and organismal levels. The systemic wound response (SWR) results in loss of tissue caused by the physical injury. In arthropods, the SWR includes a complex series of events such as the breach of basement membranes, a reactive oxygen species (ROS) burst due to the production of melanin, and the aggregation of clotting factors and hemocytes around the site of the wound (Krautz, et al., 2014). The SWR that occurs via inter-organ communication between local wound sites and remote organs ensures that the host is protected efficiently in response to a local wound. The response around the wound site is well documented, but the molecular mechanisms that allow the host to launch an organism-wide SWR are poorly understood. Following injury, studies in different model organisms reveal that infiltration of immune cells to the injury site is almost always a consequence of a rapid production of H_2_O_2_ at the site of wounding (Niethammer et al., 2009; Yoo et al., 2011; Razzell et al., 2013). This damage signal is responsible for recruiting neutrophils and other leukocytes to the wound site. ROS production causes cell death and oxidative stress, but the signaling role of ROS in injury remains unexplored. Indeed, in mammals, little information is available on the precise cellular or extracellular mechanisms by which H_2_O_2_ production promotes wound responses.

Blood cells that home to the wound site are important for clearing debris and potential pathogens from the site of injury and have also recently been shown to contribute to non-immune functions. For example, zebrafish that lack most hematopoietic tissues show defects in their capability to regenerate after amputation of the fin (Hasegawa et al., 2015). Previous studies show a link between *Drosophila* hemocytes and intestinal stem cells (ISCs), where intestinal regeneration relies on signals from hemocytes following a septic injury or intestinal epithelial damage (Ayyaz et al., 2015; Chakrabarti et al., 2016). We have shown recently that hemocytes remotely stimulate ISC proliferation following septic injury via the production of the cytokine-like secreted protein *unpaired 3* (*upd-3*; Chakrabarti et al., 2016).

Hemocyte-derived signals like upd-3 can stimulate a broad array of responses in several tissues. In larvae, following septic injury, *upd-3* is induced in hemocytes and activates JAK/STAT targets like Turandots in the fat body (Agaisse et al., 2003). In addition to stimulating mitogenesis in the gut, a recent study implicated upd-3 production from hemocytes in response to the ingestion of high-lipid-diet-regulated insulin sensitivity and lifespan (Woodcock et al., 2015). Another study involving infestation by the parasitoid wasp *Leptopilina boulardi* showed that upd-3 was released from larval hemocytes that could activate JAK/STAT signaling in the somatic muscles (Yang et al., 2015). However, we still do not understand how *upd-3* is induced after an injury.

In this study, we measured *in vivo* ROS production at the site of injury and show that activity of the NADPH oxidase Duox in hemocytes is required to increase levels of H_2_O_2_ in the vicinity of the wound. H_2_O_2_ production by and accumulation within hemocytes are essential for activation of the JAK/STAT and Toll pathways. We identify an aquaporin-like channel protein, Prip, that is required for the diffusion of H_2_O_2_ into hemocytes. We further show the importance of Toll activation following injury, as this confers protection to this subsequent infection with *Enterococcus faecalis*, indicating training of the immune response in flies. The absence of a ROS response in hemocytes during the first wound leads to a loss of the trained immune response after the secondary septic infection. Our study, therefore, underscores the central role of hemocytes in providing an integrated SWR and parallels mechanisms seen in vertebrates, where as a result of injury, infiltrating neutrophils generate ROS at the site of injury and induce innate immune responses.

## Results

### Transcriptome of Adult Hemocytes to Wounding

**Figure 1:**
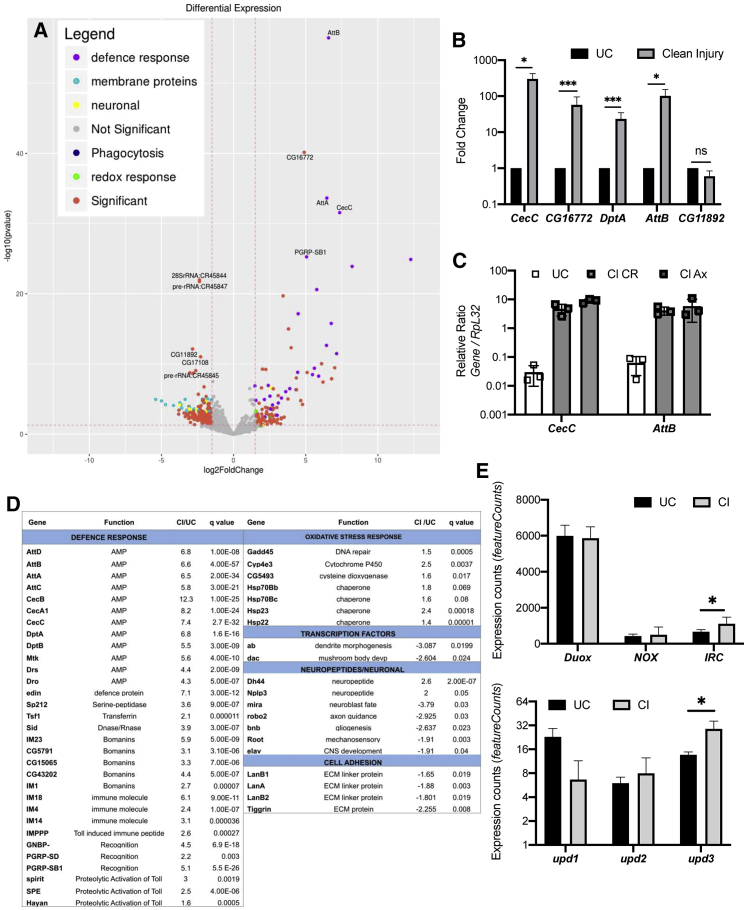
Defense Pathway Toll and Oxidative Stress Responses Are Induced upon Wounding in Hemocytes (A) A volcano plot showing genes significantly altered in hemocytes 1 h after injury. Log_2_ fold change values are plotted on the horizontal axis and −log_10_ of the p value on the vertical axis. Statistically significant regulated genes (p < 0.01) are shown in red, where GO categories are indicated (defense response, purple; membrane proteins, cyan; neuronal, yellow; redox, green; and phagocytosis, blue). (B) Validation of maximally differentially expressed genes from the RNA-seq data by qRT-PCR experiments in wild-type (*w*^*1118*^) hemocytes following injury. *CecC*, *DptA*, *AttB*, and *CG16772* were found to be significantly upregulated by qRT-PCR. ^∗∗∗^p < 0.0001; ^∗^p < 0.05; ns, non-significant. Female flies were used for experiments. (C) qRT-PCR of the AMPs *CecC* and *AttB* expression in axenic flies in comparison with conventionally reared *w*^*1118*^ flies. UC, unchallenged; CI, clean injury. (D) Differentially regulated genes from the RNA-seq experiment according to their GO categories. Gene names, functions, fold change expression, and q-values are denoted for different GO categories, including the defense response. (E) *Duox*, *NOX*, and *IRC* gene expression (top) and *upd-1*, *upd-2*, and *upd-3* gene expression (bottom) from the RNA-seq data in wild-type (*w*^*1118*^) hemocytes upon injury. *IRC* and *upd-3* were found to be significantly upregulated. ^∗^p < 0.05, as determined by Student’s t test. Counts were normalized and extracted using the software featureCounts (PMID: 24227677). ± SD are shown in (B) and (C).

To gain further insights into the mechanisms underlying hemocyte activation upon clean injury, we conducted genome-wide mRNA sequencing of hemocytes harvested from unchallenged adult flies and 1 h post-injury. We found a total of 352 genes differentially regulated with an absolute log2 fold change ≥1.5 and p value ≤ 0.05. Gene Ontology (GO) analysis revealed that the defense responsive genes, especially those dependent on Toll for their activation, emerged as the most upregulated genes after wounding (Figure 1A). We selected five most differentially expressed genes and carried out qRT-PCR in similarly isolated hemocytes. As shown in Figures 1B and S1B–S1E, the genes *CecC*, *DptA*, *AttB*, and *CG16772* were found to be significantly upregulated in response to a wound in hemocytes. *CG11892* showed an apparent decrease in expression, but this was not statistically significant (Figures 1B and S1B–S1E).

On injury microbes can enter the body cavity of flies and result in the activation of anti-microbial peptides (AMPs) in hemocytes. Therefore, we reared flies axenically and carried out qRT-PCR of hemocyte cDNA for two differentially regulated AMPs (Figure 1C) after confirming that flies were axenic by using universal bacterial 16 s rRNA gene primers on the same samples (Figure S1F). The AMPs *CecC* and *AttB* were upregulated even in axenically reared flies to levels comparable to that seen in conventionally reared flies, suggesting that Toll pathway activation was in response to injury alone (Figure 1C).

Interestingly, oxidative-stress-responsive genes were found to be significantly upregulated in the RNA sequencing (RNA-seq) data suggesting that they might be induced in response to the production of H_2_O_2_ after the wound (Figure 1D. In *Drosophila*, the NADPH oxidases produce ROS at the plasma membrane. Of the two NADPH oxidases, *Duox* expression is higher in hemocytes as compared to *NOX*, even though the levels of *Duox* remained unchanged after wounding in hemocytes, and we decided to explore the role of hydrogen peroxide increase in hemocyte activation (Figure 1E). RNA-seq analysis from injured flies also identified upregulation of the secreted, *immune-related catalase* (*IRC*) in hemocytes (Figure 1E). Therefore, our results show that there is a significant alteration in the transcriptome of hemocytes following clean injury.

### Production of H_2_O_2_ from a Wound Leads to Hemocyte Activation

**Figure 2:**
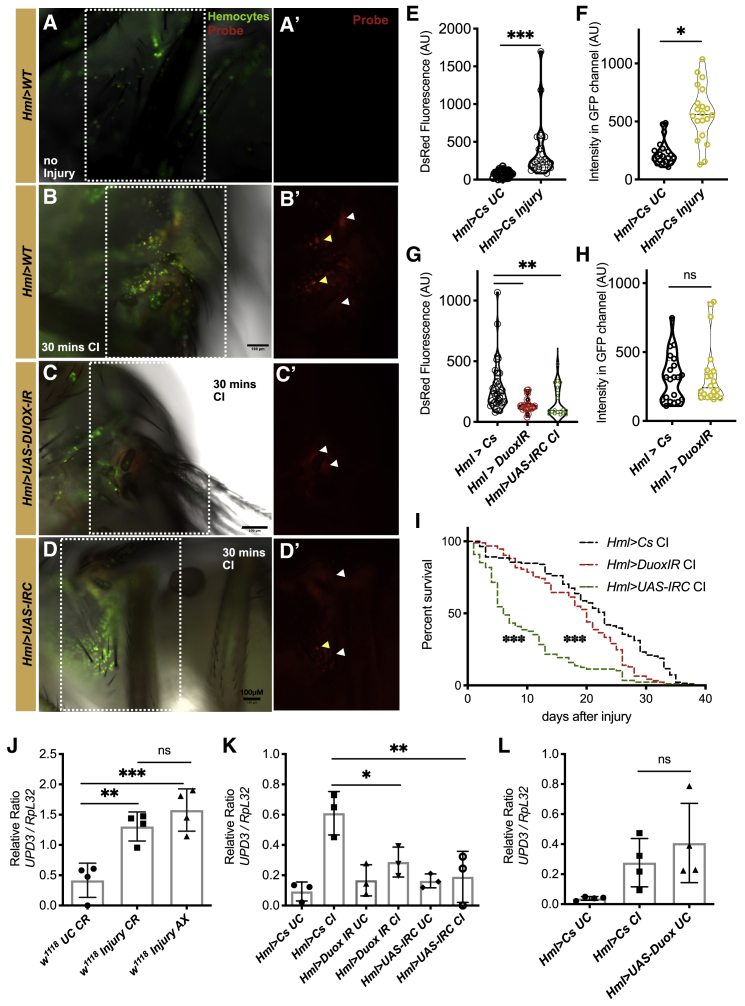
Production of Hydrogen Peroxide from a Wound Activates Hemocytes and Increases Expression of *upd-3* (A and B) Representative images of a TCFB probe (in red) before and after injury to the thorax in hemocytes using *Hml:UAS-GFP* (green) female flies. Hydrogen peroxide accumulates at the site of injury as well as in hemocytes located at that site. The TCFB probe (in red) is shown for the site of injury. (C and D) Probe fluorescence in *Hml*>*UAS-DuoxIR* flies and *Hml*>*UAS-IRC* flies within hemocytes and around the wound in the cuticle. White arrows mark cuticular ROS in (B′), (C′), and (D′), while yellow arrows mark hemocyte accumulated probe in (B′) and (D′). (E and F) Quantification of the increase in fluorescence using arbitrary units in ∼30 flies per condition and per genotype. Decreased probe fluorescence in *Hml*>*UAS-DuoxIR* (red) and *Hml*>*UAS-GFP*, *UAS-IRC* flies (green) was observed. (G and H) Quantification of the GFP fluorescence using arbitrary units in ∼20 flies per condition around the site of injury in *Hml*>*Cs* and *Hml*>*UAS-DuoxIR*. The number of hemocytes at the site of the injury was not changed upon *Duox* knockdown from hemocytes. (I) Flies with reduced ROS burst after an injury (i.e., *Hml*>*UAS-DuoxIR* and *Hml*>*UAS-IRC*) show increased susceptibility to injury. Flies per genotype from at least five independent experiments were *Hml*>*UAS-DuoxIR* (n = 93), *Hml*>*UAS-IRC* (n = 88), and wild type (*Hml*>*Cs*, n = 138). (J) Expression of *upd-3* in hemocytes in *Hml*>*UAS-DuoxIR* and *hmlΔGAL4*>*UAS-IRC* adult flies. (K) Ectopic overexpression of *Duox* in adult hemocytes is sufficient to stimulate the expression of *upd-3* in hemocytes without any injury. For (E)–(K) mean values of at least three experiments (with 30–40 flies each) ± SD are shown. ^∗∗^p < 0.01; ^∗^p < 0.05; ^∗∗∗^p < 0.001; ns, non-significant.

As H_2_O_2_ is the primary signal responsible for homing of hemocytes to the wound site in embryos (Razzell et al., 2013), we speculated that it could serve as the signal for hemocyte accumulation at the site of the wound in adult flies. We measured *in vivo* production of H_2_O_2_ through a boronate fluorescent probe (TCFB, 2-dicyanomethylene-3-cyano-4,5,5-trimethyl-2,5-dihydrofuran) (Sedgwick et al., 2017). The TCFB probe excitation wavelength is 560 nm, and emission can be collected at 580–650 nm after complexing with H_2_O_2_. Injection of this TCFB probe in the thorax of adults (which would serve as an injury), followed by imaging 30 min post-injection, revealed a significant increase in fluorescence at the site of injury (Figures 2A, 2B, and 2E). Adult hemocytes increased in number around the wound (Figure 2F), probably homing to H_2_O_2_ produced by the cuticle at the site of the injury. Interestingly, we noted significant fluorescence within hemocytes near the site of injury (Figure 2B′, yellow arrows), suggesting that hemocytes, in addition to the cuticle production of H_2_O_2_ (Figure 2B′, white arrows), also contribute to the levels of H_2_O_2_ seen on injury. Another possibility could be that hemocytes uptake H_2_O_2_ produced by the cuticle after they home to the site of injury. Probe background fluorescence and fluorescence in the absence of injury was determined by feeding the TCFB probe to female flies, as there is no way of introducing the probe into the fly without injury. In these orally fed flies, we checked both systemically and locally in the gut for background fluorescence (Figures S2A–S2C). We then infected TCFB-probe-fed flies with *Pseudomonas entomophila* to confirm that the probe had reached the gut and could report on the ROS burst that occurs after infection (Chakrabarti et al., 2012).

To confirm that hemocytes contribute to H_2_O_2_ production, Duox was knocked down specifically in hemocytes. We found that in *Duox* knockdown flies, TCFB fluorescence was significantly reduced in comparison to wild-type flies 30 min post-injury (Figures 2C, 2C′, and 2G). We did not find any change in the number of hemocytes at the injury site in *Duox* knockdown flies in comparison to the wild type (Figure 2H), and the total abundance of hemocytes in the knockdown and wild-type fat body was also comparable (Figures S2D and 3D′), indicating that cuticular ROS was sufficient for homing of hemocytes to the site of the wound. We were unable to test the role of *Duox* in the cuticle, as knockdown of this gene in the cuticle led to increased lethality even under control conditions. As RNA-seq revealed an upregulation of the catalase *IRC* in hemocytes post-injury (Figure 1E), we reduced extracellular H_2_O_2_ by overexpressing *IRC* in hemocytes. Thus, 30 min after wounding, *Hml>UAS-IRC* flies showed decreased fluorescence at the site of the wound (Figures 2D, 2C′, and 2G ) as compared to the wild-type counterpart (Figure 2B′).

**Figure 3:**
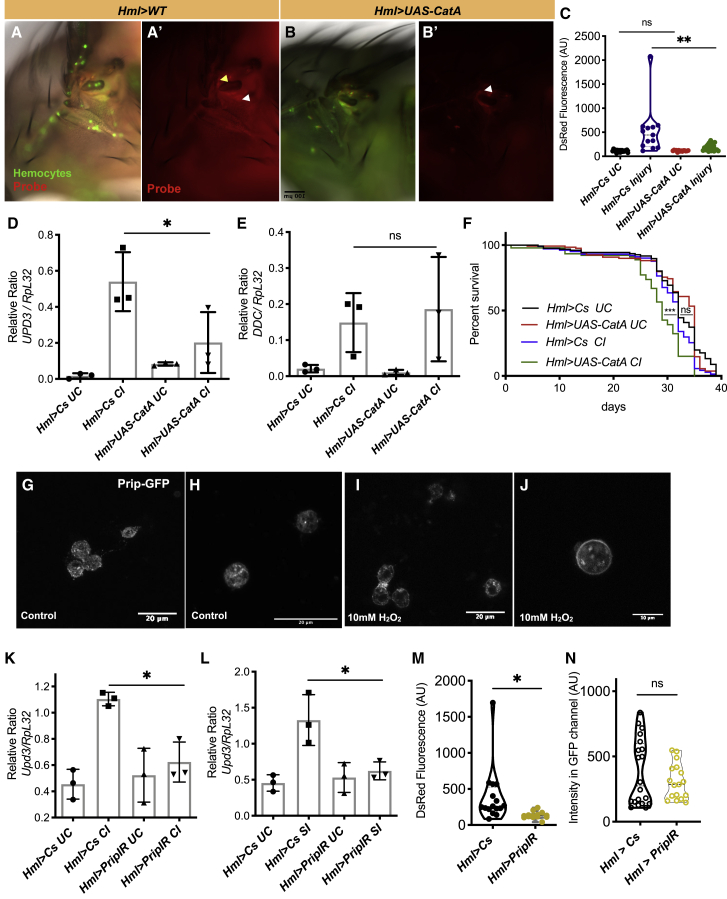
Intracellular ROS Is Required for Activation of Macrophages and Survival of Flies following Injury (A and B) Overexpression of *CatA* leads to the reduction of ROS in the cytoplasm of hemocytes. Quantification done similar to Figure 2E. White arrows mark cuticular ROS *o*n (A′), (B′), and (D′), while the yellow arrow marks hemocyte accumulated probe in (A′). (C) Decreased probe fluorescence in *Hml*>*UAS-CatA* flies upon injury as compared to their wild-type counterparts. (D and E) Expression of *upd-3* with and without injury and *ddc* expression after injury in hemocytes as compared to their wild-type counterparts (*Hml*>*UAS-CatA* versus *Hml*>*CS*). (F) Reduced intracellular ROS accumulation in hemocytes after an injury in *Hml*>*UAS-CatA* leads to increased susceptibility to injury. *Hml*>*UAS-CatA* (n = 70 [UC]; n = 76 [CI] and wild-type [*Hml*>*Cs*, n = 80 (UC); n = 70 (CI)] adult flies. UC, unchallenged; CI, clean injury. (G–J) Prip channel localization in hemocytes. Panels indicate the distribution of Prip localization in hemocytes in control media (top) and media containing 10 mM H_2_O_2_ (I and J). Membrane localization of Prip in an enlarged image is shown (J). (K) Quantification of the increase in fluorescence of the TCFB probe after an injury to the thorax in hemocytes in *prip* knockdown flies. (L) Quantification of the increase in GFP fluorescence around the wounded area in *Hml*:*UAS*-GFP animals and *prip* knockdown flies in at least 20 flies per condition. (M) qRT-PCR in wild-type flies and *Hml*>*UAS-Prip-IR*. (N) qRT-PCR in wild-type flies and *Hml*>*UAS-Prip-IR*. *upd-3* is not induced after septic injury in knocked down flies. Similar results were obtained with a second RNAi line against *Prip-IR* line (BDSC_50695). ^∗∗^p < 0.01; ^∗^p < 0.05; ns, non-significant. ± SD are shown in (D), (E), (M), and (N).

Next, we asked if the ROS burst contributed to host survival after an injury. RNAi-mediated knockdown of *Duox* in hemocytes led to an increased susceptibility to both a clean injury (Figures 2I and S2E) as well as septic injury with the gram-negative bacterium *Ecc15* (Figure S2F). Importantly, knockdown of *Duox* in hemocytes did not adversely affect longevity under control conditions (Figure S2F). In a manner similar to the knockdown of *Duox*, the overexpression of *IRC* increased the susceptibility of flies to succumb to wounding as well as septic injury (Figures 2I and S2F). In conclusion, using recently developed H_2_O_2_ probes *in vivo*, we were able to measure the ROS burst after an injury in the adult (Figures 2A–2E) and show that hemocyte ROS production is critical for the survival of injured flies.

### Injury-Induced ROS Burst Is Essential for *upd-3* Expression

We have shown earlier that upd-3 produced from hemocytes activates JAK/STAT signaling in the adult fly gut, which is crucial for survival following a septic injury in adult flies (Chakrabarti et al., 2016). Since upd-3 was induced on wounding in hemocytes, we knocked down both *upd-3* and its close homolog, *upd-2*, in hemocytes and saw an increased susceptibility to a simple injury (Figure S2G). These knocked down flies were also susceptible to infection with *Ecc15* (Figure S2H). Hence, the production of these ligands from hemocytes post-injury or infection is essential for the survival of flies under these two stresses.

On wounding, cuticular microbes may enter the body cavity, and their microbial patterns might be recognized by hemocytes, thereby contributing to the expression of *upd-3*. We therefore isolated hemocytes from injured axenically reared flies and monitored *upd-3* expression. Surprisingly, *upd-3* induction was the same in hemocytes on wounding in germ-free flies when compared to conventionally reared animals (Figure 2J). Therefore, the induction of *upd-3* in hemocytes was primarily due to damage caused by the injury and not pathogens that might breach the cuticle and enter circulation.

Next, we investigated the link between *upd-3* induction in hemocytes and ROS production upon injury. We reduced the levels of ROS in flies using the *Hml>DuoxIR* and *Hml>UAS-IRC* lines and monitored the induction of *upd-3*. Both sets of flies showed an attenuated induction of *upd-3* after an injury (Figure 2K). Similar results for reduced *upd-3* induction were seen with a septic injury in the two fly lines (Figure S3A). Therefore, reduced *upd-3* production by flies with lower ROS levels produced by hemocytes could contribute to the increased susceptibility of these flies to injury.

A reduction in ROS levels from hemocytes could negatively impact all wounding-related genes in hemocytes. To test this, we choose *ddc*, another wound-induced gene, which is essential in the melanization reaction upon injury and is induced via the transcription factor *Grainy-head* (Kim and McGinnis, 2010). We found no change in *ddc* expression in the fly lines *Hml>DuoxIR* and *Hml>UAS-IRC* (Figure S3B). In addition, we validated the efficiency of the knockdown of *Duox* (Figure S3C) and used another independent hemocyte driver line (*pxn-Gal4*) and RNAi line against *Duox* to confirm that reduced *Duox* expression in hemocytes decreased the induction of *upd-3* from hemocytes after an injury (Figures S3D and S3E). To confirm whether RNAi conditions impacted hemocyte numbers in general, which could then lead to a reduction of *upd-3* expression, we normalized the qPCR data to hemocyte-specific genes. Consistent changes in gene expression were observed even after normalization with *hemese* and *hemolectin* (Figures S3F and S3G).

To investigate whether the enhanced ROS levels were sufficient to induce *upd-3*, we used the hemocyte driver *Hml* to acutely overexpress *Duox* and monitored the induction of *upd-3* as a readout of the JAK/STAT pathway and observed that *upd-3* was induced in the absence of injury (Figure 2L). Taken together, our results show that H_2_O_2_ produced after an injury was both necessary and sufficient for the induction of *upd-3* by hemocytes in adult *Drosophila*. Therefore, the ROS burst is a danger-associated molecular pattern (DAMP) that leads to the activation of hemocytes after a wound.

### Intracellular ROS Contributes to the Activation of Macrophages

An increase in TCFB probe fluorescence was observed within hemocytes that were close to the wound site (Figures 2A and 2A′). It has previously been demonstrated that ROS levels increase in the epithelia around a wound in both *Drosophila* and zebrafish embryos (Hunter et al., 2018). Appropriate levels of ROS in tissues are maintained by a series of enzymes, including superoxide dismutase (Sod) and catalase (Nordberg and Arnér, 2001). To explore the role of intracellular H_2_O_2_, we first expressed a fly cytosolic catalase, CatA, in hemocytes and monitored the impact of ROS levels. The levels of ROS detected at the site of the wound were attenuated on overexpressing *CatA* in hemocytes (Figures 3A–3C). Furthermore, overexpressing *CatA* in hemocytes reduced *upd-3* mRNA levels (Figure 3D) and increased susceptibility to wounding (Figure 3F). No reduction was seen in the expression of *ddc* after overexpressing CatA (Figure 3E).

What is the source of this intracellular H_2_O_2_ within hemocytes? ROS could be produced by mitochondria within hemocytes or enter hemocytes through diffusion from the surrounding epithelia (Figure S4A). In addition to manipulating the levels of a catalase, we overexpressed the mitochondrial superoxide dismutase Sod2, known to act as a mitochondrial ROS scavenger, in hemocytes (Sinenko et al., 2011). To lower levels of cytoplasmic ROS, we overexpressed cytoplasmic Sod1 within the hemocyte. We wounded Sod1- and Sod2-overexpressing flies and observed that only Sod1 overexpression reduced *upd-3* induction in hemocytes (Figure S4B). This indicates that the cytoplasmic accumulation of H_2_O_2_ in hemocytes does not result from mitochondrial ROS generation. No induction of *ddc* after overexpressing both Sod1 and Sod2 (Figure S4C) was observed. Sod1 overexpression increased the susceptibility of flies to injury (Figure S4D) while having little effect on their longevity under control conditions (Figure S4E). In summary, accumulation of H_2_O_2_ in *Drosophila* blood cells at the site of injury is essential for *upd-3* activation and increased longevity of injured flies.

### Aquaporin-Mediated ROS Uptake Is Critical for Hemocyte Activation

Having shown that intracellular ROS is required for upd-3 production by hemocytes, we wondered if H_2_O_2_ enter hemocytes through diffusion and/or facilitated transport. For example, H_2_O_2_ could accumulate in hemocytes by passive diffusion from the hemolymph. Alternatively, facilitated diffusion could be mediated by membrane transporters. Intriguingly, such transporters have not been identified thus far in hemocytes. The extracellular catalase IRC was found to be upregulated in hemocytes on wounding (Figure 1E), and further overexpression attenuated the induction of *upd-3* (Figure 2K). This suggested that extracellular H_2_O_2_ may contribute to the accumulation of intra-hemocyte ROS, in addition to that produced within the cells.

In mice, chemokine-dependent migration of T cells requires the expression of aquaporin 3 (AQP3) and its ability to transport H_2_O_2_ into T cells (Hara-Chikuma et al., 2012). A recent report demonstrated that in mammalian intestinal epithelial cells, AQP3 facilitates not only water but also H_2_O_2_ movement into these cells (Thiagarajah et al., 2017). Interestingly, on curating a list of the most upregulated genes in hemocytes in response to infection, as well as genes unregulated in *Hop*^*tum-l*^ mutant larvae that show constitutive JAK/STAT signaling (Irving et al., 2005), we identified an aquaporin homolog, Prip, to be highly induced. The fly aquaporin protein Prip is most closely related to the mammalian channel AQP1, with ∼40% identity (Figure S5A). AQP1 facilitates the transport of H_2_O_2_ into cells, and modeling the channel pocket of Prip alongside the known structure of AQP1 (PDB: 1J4N) showed that there was a high degree of similarity between the channels (Figure S5A). We therefore asked if this structural similarity could result in functional conservation, whereby Prip could transport H_2_O_2_ into hemocytes following injury.

We generated a transgenic line where Prip was tagged at its N terminus with a superfolder GFP tag. We found that Prip-GFP was distributed in what appear as vesicles in the cytoplasm of hemocytes as punctae with enrichment at the membrane on addition of H_2_O_2_ (Figures 3G–3J). To explore the role of Prip after injury, we knocked down *Prip* in hemocytes and monitored *upd-3* induction. Indeed, decreasing *Prip* expression in hemocytes reduced *upd-3* mRNA levels (Figure 3K) and the levels of ROS detected at the site of the wound, without a change in the recruitment of hemocytes at the wound site (Figures 3M and 3N). Similarly, reduced *Prip* expression by hemocytes led to decreased induction of *upd-3* following septic injury (Figure 3L), with no effect on *ddc* induction (Figure S5C).

To test if Prip transports H_2_O_2_, HEK cells were preloaded with TCFB and transfected with *Drosophila* Prip channel. Prip-overexpressing HEK293E cells showed a greater relative increase in intracellular fluorescence compared to control cells upon addition of 100 μM H_2_O_2_ (Figures S5B and S5B′). In summary, transport of H_2_O_2_ into *Drosophila* blood cells at the site of injury and accumulation of intracellular ROS are essential for hemocyte activation and increased longevity of injured flies. Therefore, Prip plays a key role in increasing intra-hemocytic ROS levels, enabling immune activation of hemocytes upon injury.

### Induction of upd-3 Is Dependent on the Src42A/Shark/Draper Pathway

**Figure 4:**
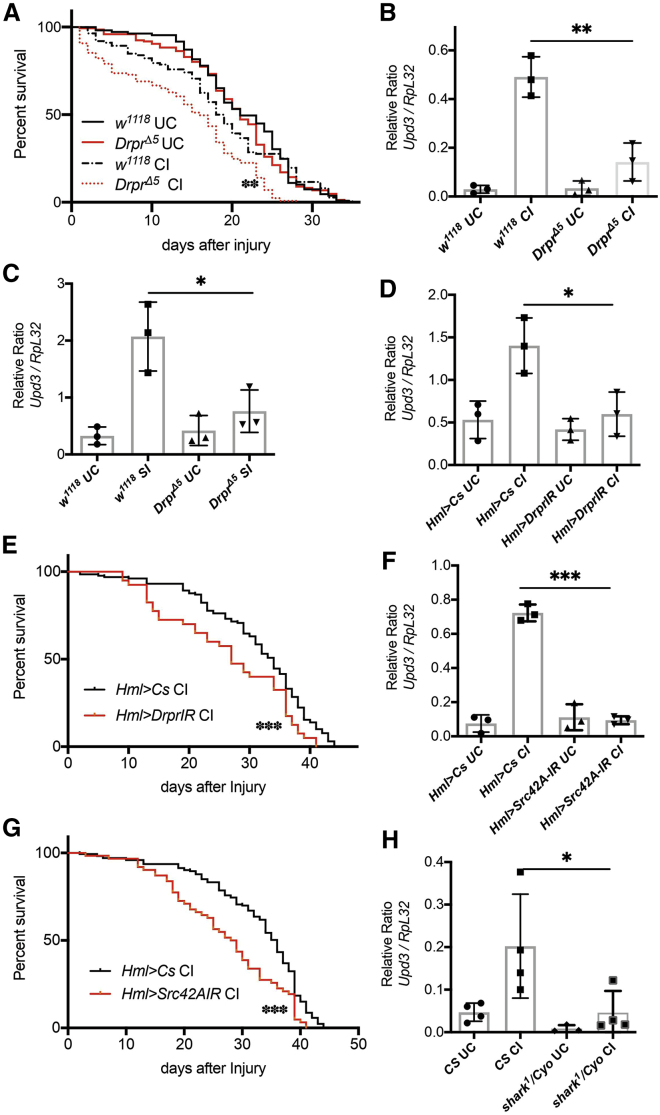
The Src42A/Shark/Draper Pathway Regulates *upd-3* Expression in Hemocytes (A, E, and G) Lifespan of *drpr*^*Δ5*^ flies and susceptibility to injury as compared to the wild type (*w*^*1118*^). Flies per genotype are pooled from at least three independent experiments. Log-rank test was used for comparing wild-type (*w*^*1118*^; CI, n = 112; SI, n = 63) and *drpr*^*Δ5*^ (CI, n = 129; SI, n = 61) flies; *Hml*>*UAS-drprIR* (n = 81) and *Hml*>*UAS-Src42AIR* (n = 62) adult flies as compared to wild type (*Hml*>*Cs*, n = 173). UC, unchallenged; CI, clean injury; SI, septic injury. (B and C) qRT-PCR of *upd-3* in *drpr*^*Δ5*^ mutant flies 1 h after injury (B) and septic injury (C). (D, F, and H) Hemocyte knockdown of *drprIR* (D), *src42AIR* (F), and *shark*^*1*^ heterozygotes (H) shows a decreased expression of *upd-3* expression 1 h after injury (±SD is shown). ^∗∗∗^p < 0.0001; ^∗∗^p < 0.01; ^∗^p < 0.05; ns, non-significant.

The migration of embryonic hemocytes to sites of damage where H_2_O_2_ is produced is regulated by the apoptotic receptor Draper (drpr), together with the Src family kinase Src42A and a Syk homolog, Shark (Figure S4A) (Evans et al., 2015). H_2_O_2_ drives wound closure through Src42A, which promotes polarization of junctions and cytoskeleton around wounds (Hunter et al., 2018). A previous study demonstrated that homozygous *drpr*^*Δ5*^ adults have reduced longevity (Draper et al., 2014). We therefore backcrossed *drpr*^*Δ5*^ flies for nine generations and found that they showed no change in their longevity when compared to wild-type flies (Figure 4A). We monitored induction of *upd-3* cytokine in adult hemocytes in these backcrossed flies. Similar to the effect seen on reduction of ROS production after an injury, deletion of *drpr* led to an increased susceptibility of flies to both clean and septic injury (Figures 4A and S6A). Concomitantly, *drpr*^*Δ5*^ knockout flies showed a significant decrease in *upd-3* induction in hemocytes after an injury (Figure 4B) while showing no change in *ddc* expression (Figure S6B). *drpr*^*Δ5*^ mutant flies also showed a lack of *upd-3* induction after a septic injury (Figure 4C), while *ddc* expression showed a similar induction in mutant flies (Figure S6C).

Draper is expressed not only by hemocytes but also by glial cells (Freeman et al., 2003). We therefore knocked down *drpr* exclusively in hemocytes and continued to observe a decrease both in the induction of *upd-3* and susceptibility to injury (Figures 4D and 4E). Knockdown of *drpr* led to a 2-fold decrease in *drpr* transcripts (Figure S6D), and flies showed a significant decrease in *upd-3* induction after septic injury with no change in *ddc* (Figures S6E and S6F).

Draper is phosphorylated *in vitro* by the fly homolog of the Lyn kinase, Src42A (Logan et al., 2012). Draper and Src42A have been shown to interact genetically in embryonic hemocytes (Evans et al., 2015). In zebrafish, Lyn is redox sensitive in neutrophils (Yoo et al., 2011). To test whether Src42A is responsive to H_2_O_2_ in adult hemocytes, we knocked down *src42A* specifically in the hemocyte and observed a decrease in *upd-3* induction and increased susceptibility after an injury (Figures 4F and 4G). Furthermore, all consequences of injury alone were conserved following septic injury in *src42A* knockdown flies (Figures S6E and S6F).

Src42A and the downstream kinase Shark have been shown to regulate the homing of embryonic hemocytes to H_2_O_2_ after a laser-induced wound (Evans et al., 2015). Hence, *shark*^*1*^ heterozygote adults were injured, and the induction of *upd-3* was monitored 1 h after the injury. We observed lower induction of *upd-3* after an injury, with no change in levels of *ddc* in *src42A* RNAi animals or *shark*^*1*^ heterozygote adults (Figures S6G and S6H). Therefore, Src42A/Shark/Draper, in addition to their roles in allowing hemocytes to migrate toward a wound and phagocytose apoptotic corpses in the embryo, are important in adult hemocytes for *upd3* induction (Evans et al., 2015; Weavers et al., 2016).

### The Toll Defense Pathway Is Induced in Response to ROS upon Wounding

We found Toll pathway genes were induced in hemocytes on injury, as seen in RNA-seq analysis (Figure 1B). To determine if Toll signaling is active in hemocytes, we monitored nuclear dorsal localization in hemocytes in injured flies, using the anti-dorsal 7A4 antibody. Under unchallenged conditions dorsal was cytosolic, while wounding led to nuclear localization of dorsal in hemocytes (Figures S7A and S7A′). We then decided to knock down the Toll1 receptor specifically in hemocytes with the *hml* driver. Survival analysis show that Toll pathway activation in hemocytes was critical for the survival of flies following injury (Figure S7B). We next addressed whether heightened ROS levels were sufficient to induce Toll target genes without any injury. We therefore used the hemocyte driver *Hml* to acutely overexpress *Duox* and monitored the induction of the AMP *CecC*. *Cec* was indeed induced in the absence of injury (Figure S7C). Hence, in addition to activating the JAK/STAT ligand *upd-3*, the ROS burst produced after a wound causes the activation of Toll signaling in hemocytes, and H_2_O_2_ production by hemocytes, even in the absence of injury, is sufficient to turn on the Toll pathway (Figure S7A)

### Injury Confers Protection against *E. faecalis* Systemic Infection

**Figure 5:**
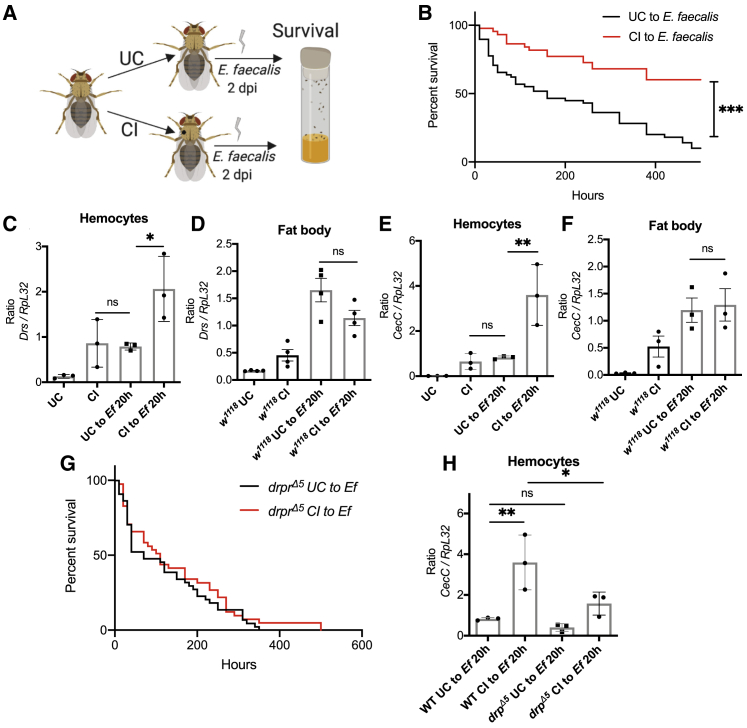
Injury Protects Flies from a Subsequent Systemic Infection (A) Experimental setup for determining immune training after an injury to a pathogen systemic infection. Flies were either injured or left undisturbed prior to infection with *E. faecalis*. (B) Survival of flies following infection with *E. faecalis* as indicated in (A) (*w*^*1118*^; UC, n = 58; CI, n = 44). (C–F) qRT-PCR of the AMPs *CecC* and *drosomycin* (*drs*) expression in hemocytes and fat body of naive versus injured flies *w*^*1118*^ flies that were subsequently infected with *E. faecalis* for 20 h. (G) Survival of *drpr*^*Δ5*^ mutant flies with *E. faecalis* as indicated in (A). (H) qRT-PCR in wild-type and *drpr*^*Δ5*^ mutant flies in hemocytes of naive versus injured flies that were subsequently infected with *E. faecalis* for 20 h. The loss of response to the ROS signal upon wounding in leads to an attenuated hemocyte AMP activation to infection with *E. faecalis*. UC, unchallenged; CI: clean injury; *Ef*, *E. faecalis*. For (C)–(F) and (H), data represent ±SD. ^∗^p < 0.05; ns, non-significant.

To determine whether activation of the immune pathways post-injury provides memory toward a subsequent infection, we systemically infected flies with a gram-positive bacterium *E. faecalis*. We observed that flies wounded 2 days prior to a systemic infection showed a dramatically improved survival as compared to their naive counterparts (Figures 5A and 5B). This suggests training of the innate immune system in flies. To further explore how long this immune training is maintained, we injured flies and subsequently infected them with *E. faecalis* 5 and 7 days after the first injury. We found that at 5 days post-injury (dpi), flies showed a delay in the death rate with an increase in maximum lifespan, whereas at 7 dpi, there was no advantage conferred to the injured flies when compared to their naive counterparts (Figures S7D and S7E).

Toll pathway genes such as AMPs are induced in hemocytes on systemic infection with *E. faecalis*. We therefore asked if there was a heightened response in hemocytes in previously injured flies compared to their naive counterparts that could explain the protection we observed (Figure 5B). We carried out qRT-PCR of hemocyte RNA for two differentially regulated anti-microbial peptides, *drs* and *CecC* (Figures 5C and 5E), and found that injured flies showed increased AMP induction on subsequent infection with *E. faecalis*. In addition to AMP production by hemocytes, the fat body could contribute to protection in previously injured flies. We therefore measured AMP induction in the fat body but found no significant difference in AMP induction following *E. faecalis* systemic infection between injured flies and naive flies (Figures 5D and 5F).

In order to assess whether immune training following injury was dependent on the ROS burst after the wound, we infected *drpr*^*Δ5*^ flies where the response to ROS was abrogated in hemocytes. The *drpr*^*Δ5*^ mutant flies did not show protection in survival analysis when pre-injured to *E. faecalis* infection (Figure 5G). In infected *drpr*^*Δ5*^ flies, we monitored the activation of the AMP *CecC* on septic injury. *drpr*^*Δ5*^ flies failed to show enhanced *CecC* activation on a subsequent *E. faecalis* infection while having a comparable AMP induction under naive systemic infection (Figure 5H). Furthermore, to determine if the ROS response that activated hemocytes to an injury was crucial for this increased AMP induction on a subsequent challenge, we temporally overexpressed SOD1 in hemocytes just prior to the first injury. Subsequently, these flies were raised under restrictive conditions to achieve wild-type levels of ROS. Flies that did not elevate intracellular ROS levels after an injury failed to show a heightened *CecC* activation to a second *E. faecalis* infection (Figure S7F). In line with this observation, these SOD1-overexpressing flies, when injured, were not protected against a second systemic infection with *E. faecalis* (Figure S7G) when compared to their wild-type counterparts.

**Figure 6:**
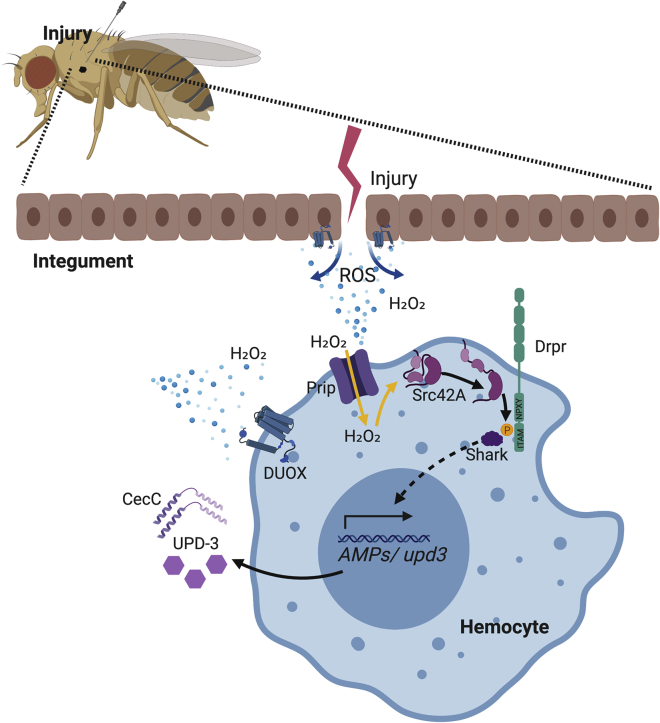
Features of a Response to Systemic Injury in Flies Following injury, hemocytes respond to the damage signal H_2_O_2_, which is produced by the NADPH oxidase Duox, through the kinase Src42A and its downstream targets Shark and Draper. A channel protein, Prip, helps increase intracellular ROS levels within hemocytes to trigger the Toll and JAK/STAT signaling pathways, contributing to trained immunity.

In conclusion, we have shown here that hemocytes play a crucial role in providing an integrated SWR to ensure homeostasis of the whole organism (Figure 6). In the absence of this response, a simple injury becomes deleterious to the organism and leads to its early death. Following an injury, hemocytes activate an inflammatory program similar to that which occurs upon infection, providing protection from a subsequent systemic infection and serving as evidence of trained immunity in flies.

## Discussion

In *Drosophila* and other arthropods, hemocytes help mediate wound healing through melanization that is important for clot formation and production of cytotoxic intermediates that kill invading microorganisms (Krautz et al., 2014). Our results show an induction of oxidative stress genes possibly in response to H_2_O_2_ accumulation in the hemocytes after wounding (Figure 1D). One such gene that was significantly upregulated and is involved in wound healing in embryos is Gadd45 (Figure 1D; Stramer et al., 2008). We also found small Hsps upregulation upon wounding, particularly in sHsp22 which has been linked to protection against oxidative stress (Morrow et al., 2004). These chaperones might be needed to deal with the damage caused by an increase in ROS levels in hemocytes after a wound.

RNA-seq of activated hemocytes revealed a number of additional genes that were mis-regulated. For example, expression of transcription factors involved in neuron specification and differentiation, such as *abrupt* (*ab*) and *dachshund* (*dac*) (Yasugi and Nishimura, 2016), were increased. In mammals, there is an overlap between the genetic programs of hematopoietic and neural stem cells (Terskikh et al., 2001). In flies, the transcription factor glial-cells-missing (gcm) is the primary regulator of glial cell fate as well as differentiation of hemocytes, and *gcm* and *gcm2* mutant flies show a marked reduction of hemocyte numbers (Bernardoni et al., 1997; Alfonso and Jones, 2002). Therefore, ab and dac may also function in hemocyte differentiation and activation.

In mammals, cutaneous injury results in H_2_O_2_ production and contributes to wound healing by promoting wound closure and angiogenesis (Roy et al., 2006). In addition, H_2_O_2_ is vital for enhanced epithelial cell migration in response to injury in the lung and intestine (Gorissen et al., 2013; Jones et al., 2013). We show here that ROS production in hemocytes through the NADPH oxidase Duox is critical for the survival of flies following injury and that ectopic Duox induction from hemocytes is sufficient to induce upd-3. In mammalian airway epithelia, DUOX1 contributes to the induction of matrix metalloproteinase 9 and the neutrophil chemokine interleukin IL-8 (CXCL8) via the ERK1/2 and nuclear factor κB (NF-κB), pathways, which are involved in the wound-healing program (Koff et al., 2008). The synthesis of cytokines such as CXCL8 after injury can affect macrophage activation and furthermore induce neutrophil influx to the site of injury (Forman and Torres, 2002).

Aquaporins are a group of channels that transport water, glycerol, or H_2_O_2_ across various cell types (Verkman, 2011). AQP1 is expressed in activated B and T lymphocytes, as well as lymphocytes surrounding infiltrating tumors (Moon et al., 2004). The exact role of AQP1 in the activation and migration of these immune cells to tumors remains to be determined. AQP1 is required for the migration of endothelial cells, while AQP4 in astrocytes is polarized to the leading edge of migrating cells, facilitating lamellipodial extension and increasing cell migration (Verkman, 2011). It appears that the *Drosophila* AQP1 homolog Prip is important for the buildup of intra-hemocyte ROS and activation of hemocytes after injury (Figures 3K–3N). It would now be interesting to follow Prip-deficient hemocytes to study the role of this aquaporin in migration after wounding.

Studies with zebrafish have shown the importance of the Src family kinase Lyn in tail fin regeneration after injury (Yoo et al., 2011). H_2_O_2_ dependent activation of Lyn by oxidation of several cysteine residues is essential for the migration of neutrophils toward the site of injury. Src42A, the *Drosophila* Lyn homolog, is also required for hemocyte migration toward wounds (Evans et al., 2015). We demonstrate here that H_2_O_2_ is the signal that activates the Src42A\Shark\Draper pathway in adult hemocytes.

Similar to mammals, our study demonstrates that the Toll pathway can be activated in hemocytes in the absence of any pathogens or microbes. The Toll pathway has been linked to resistance against septic injury and is also activated in the fat body of larvae upon muscle damage (Green et al., 2018). In fly larvae, wasp parasitism increased ROS levels in the lymph gland and led to activation of Toll and epidermal growth factor receptor (EGFR) signaling in the hematopoietic lymph gland progenitors, which are required for timely lamellocyte differentiation (Louradour et al., 2017). The role of the Toll pathway in a sterile inflammatory reaction has relevance to mammalian physiology, since gain-of-function mutations in the Toll-like receptor (TLR) signaling pathway in humans are associated with many hematological malignancies (Wang et al., 2014). Interestingly, in models of cell competition where fitter wild-type neighbors eliminate pre-malignant cells, the Toll pathway prevents apoptosis in mutant cells, thereby converting them to super-competitors (Katsukawa et al., 2018).

It is becoming increasingly clear that wound-healing pathways overlap with pathways that are activated in cancer cells. Studies with flies have revealed that a localized immune response by hemocytes is necessary to control the growth of neoplastic tissue (Pastor-Pareja et al., 2008; Parisi et al., 2014). In flies, Fogarty et al. (2016) recently linked Duox-produced ROS from tumor cells to the recruitment of hemocytes, which are activated to secrete the tumor necrosis factor (TNF) ortholog. It is worth mentioning that chemotherapy leaves patients with a loss of neutrophils (Kuderer et al. 2006). As neutrophils share some features with hemocytes, the delineation of signaling pathways in the hemocyte that we describe here could provide clues as to how neutrophils control cancer progression.

In invertebrates and plants, the innate immune system has an ability to “retain memory” for a few weeks to months (Netea et al., 2016). For example, a primary infection of mosquitoes with *Plasmodium* can induce innate immune memory protection for mosquitoes against a subsequent *Plasmodium* infection through the action of their gut microbiota (Rodrigues et al., 2010). This trained immune response relies on an increased number circulating hemocytes and an enhanced response by hemocytes to a secondary infection. In the context of our study, damage-induced inflammation could be a signal to train the immune cells of *Drosophila* so as to provide an improved defense response when reinfected with a real pathogen. One of the mechanisms for this trained immune response could be a sustained upregulation of immune regulatory pathways such as Toll in the hemocytes and consequent phenotypic changes in immune cell populations. Recent studies have highlighted the concept of trained immunity in mammals, as seen in natural killer (NK) cells and macrophages (Saeed et al., 2014; Netea et al., 2016). The mechanisms underlying this training remain unknown, and therefore, *Drosophila* could provide a model system to identify important molecular players in this trained immune response.

The activation of inflammatory pathways in the absence of infection after an injury might shape the immune activation of the same hemocytes in subsequent challenges. Here, we show that a previous injury does indeed confer an advantage to flies to a bacterial systemic infection. The inflammatory response mounted by hemocytes involving activation of the Toll pathway to an injury alone may account for this survival benefit. In mammals, epigenetic reprogramming occurs in NK cells and dendritic cells after an initial response that changes immune cell physiology, leading to protection to a subsequent infection (Netea et al., 2016). Our study shows that hemocytes display memory in terms of Toll activation after an injury that confers an advantage to a subsequent pathogen attack. In the absence of ROS signaling in hemocytes after the first injury, these hemocytes fail to mount a protective effect on a second infection. In mammals, similar training of cytokine production by innate immune cells has been described to be induced by endogenous DAMPs (Crișan et al., 2016). The signaling pathways and cytokines that mediate inflammatory response to injury are amenable to analysis by mutagenesis screening approaches, which will make *Drosophila* a valuable tool in deciphering mechanisms that bring about trained immunity.

## STAR★Methods

### Key Resources Table

**Table:** 

REAGENT or RESOURCE	SOURCE	IDENTIFIER
**Antibodies**		

Mouse anti-dorsal	DSHB	7A4, RRID: AB_528204
Goat anti-mouse IgG (H+L) Cross-absorbed Secondary Antibody, Alexa Fluor 488	ThermoFisher	Cat# A-11008, RRID: AB_143165

**Bacterial and Virus Strains**		

*Erwinia carotovora carotovora15*	Basset et al., 2000	N/A
*Enterococcus faecalis*	Dr. Alejandro Aballay, OSHU	OG1RF

**Chemicals, Peptides, and Recombinant Proteins**		

Dextrose	Sisco Research Laboratories Pvt Lt	Cat# 51758
European Bacto Agar	CondaLab	Cat# 1800
Yeast extract	Sisco Research Laboratories Pvt Lt	Cat# 34266
LB Broth	CondaLab	Cat# 1231
DNase I	ThermoFischer Scientific	Cat# EN0521
Trizol (RNAiso Plus)	TaKaRa Bio	Cat# 9108
Revertaid reverse transcriptase	ThermoFischer Scientific	Cat# EP0441
SYBR Green	TaKaRa Bio	Cat# RR420A
TCFB probe (boronate of 2-dicyanomethylene-3-cyano-4,5,5-trimethyl-2,5-dihydrofuran	Dr Chakrapani	Sedgwick et al., 2017
DPBS	Sigma	Cat# D5652
Tween 20	ThermoFischer Scientific	Cat# 10419000
4’,6- diamidino-2-phenylindole DAPI	Sigma-Aldrich	Cat# D9542
Formaldehyde	Sigma-Aldrich	Cat# SHBG2949V
2% n-propyl gallate	Sigma-Aldrich	Cat# P3130
Bovine Serum Albumin (BSA)	Sigma-Aldrich	Cat# A3059-10G

**Critical Commercial Assays**		

Direct-zol RNA kit	Zymo research	Cat#R2051
NEBNext Ultr II RNA Library Prep Kit for Illumina	NEB	Cat#E7770S
NEB Next Poly(A) mRNA Magnetic Isolation Module	NEB	Cat#E7490S

**Deposited Data**		

Raw and analyzed data	This paper	ArrayExpress: E-MTAB-8090

**Experimental Models: Cell Lines**		

Human: Human embryonic kidney (HEK) 293E	ATCC	CRL-1573

**Experimental Models: Organisms/Strains**		

*D. melanogaster*: RNAi of Upd2: *w;UAS-upd2-IR*	Kyoto Stock Center	NIG:5988,R3
*D. melanogaster*: RNAi of Upd3: *w;UAS-upd3-IR*	Bruno Lemaitre	Agaisse et al., 2003
*D. melanogaster*: *hml*Δ*Gal4,UAS-GFP* (*w1118; P{w+mC = Hml-GAL4.G}6-4 P{w+mC = UAS-GFP::lacZ.nls}15.1)*	Bruno Lemaitre	Sinenko et al, 2011
*D. melanogaster*: RNAi of Duox: *UAS-Duox-IR*	Bruno Lemaitre	Ha et al., 2005
*D. melanogaster*: *UAS-IRC*	Bruno Lemaitre	Ha et al., 2005
*D. melanogaster*: RNAi of Duox: *UAS-Duox-IR-2* (*y[1] sc[^∗^] v[1]; P{y[+t7.7] v[+t1.8] = TRiP.HMS00934}attP2*)	Bloomington Drosophila Stock Center	RRID: BDSC_33975
*D. melanogaster*: *UAS-CatA* (*w[1]; P{w[+mC] = UAS-Cat.A}2*)	Bloomington Drosophila Stock Center	RRID: BDSC_24621
*D. melanogaster*: *pxn-Gal4*	Bruno Lemaitre	Stramer et al., 2005
*D. melanogaster*: RNAi of Src42A: *w;UAS-src42A-IR*	Kyoto Stock Center	NIG:7873,R3
*D. melanogaster*: *shark*^*1*^ (*P{ry[+t7.2] = neoFRT}43D Shark[1]/CyO*)	Bloomington Drosophila Stock Center	RRID: BDSC_5865
*D. melanogaster*: RNAi of Drpr: *w;UAS-drpr-IR* (*y[1] sc[^∗^] v[1] sev[21]; P{y[+t7.7] v[+t1.8] = TRiP.HMS01623}attP2*)	Bloomington Drosophila Stock Center	RRID: BDSC_36732
*D. melanogaster*: *drpr*^*Δ5*^	Dr T. Kuraishi	Freeman et al., 2003
*D. melanogaster*: RNAi of Prip: *UAS-Prip-IR* (*y[1] sc[^∗^] v[1]; P{y[+t7.7] v[+t1.8] = TRiP.GLC01619}attP2*)	Bloomington Drosophila Stock Center	RRID: BDSC_44464
*D. melanogaster*: RNAi of Prip: *UAS-Prip-IR* (*y[1] v[1]; P{y[+t7.7] v[+t1.8] = TRiP.HMC03097}attP40*)	Bloomington Drosophila Stock Center	RRID: BDSC_50695
*D. melanogaster*: *UAS-Sod1* (w[1]; P{w[+mC] = UAS-Sod1.A}B37)	Bloomington Drosophila Stock Center	RRID: BDSC_24750
*D. melanogaster*: *UAS-Sod2* (*w*^*1*^*; P{UAS-Sod2.M}UM83*)	Bloomington Drosophila Stock Center	RRID: BDSC_24494
*D. melanogaster*: RNAi of Toll: *w;UAS-Toll-IR* (*y1 v1; P{TRiP.JF01276}attP2*)	Bloomington Drosophila Stock Center	RRID: BDSC_31477
*D. melanogaster*: Fosmid line of Prip: *y[1], w[^∗^]; PBac{y+,FlyFos.Prip-GFP.S}*	This paper	N/A
*D. melanogaster*: *w1118* (wild type)	Bruno Lemaitre	N/A
*D. melanogaster*: *CantonS* (wild type)	Bruno Lemaitre	N/A

**Oligonucleotides**		

Quantitative PCR primers, see Table S1	This paper	N/A
NEBNext Multiplex Oligos for Illumina	NEB	NEB, Cat#E7335S

**Recombinant DNA**		

Prip fosmid tagged with sGFP-V5-BLRP-FLAG	Source BioScience	FlyFos029594
cDNA LD27313	Drosophila Genomics Resource Center	DGRC:2038; FlyBase:FBcl0176667
pcDNA-Prip	This paper	N/A

**Software and Algorithms**		

ImageJ (Fiji)	Schindelin et al., 2012	https://fiji.sc/
EdgeR	McCarthy et al., 2012; Robinson et al., 2010	https://www.bioconductor.org/packages/release/bioc/html/edgeR.html
FASTQC	Babraham Bioinformatics	https://www.bioinformatics.babraham.ac.uk/projects/fastqc/
STARv2	Dobin et al., 2013	https://github.com/alexdobin/STAR
FeatureCounts in R	The R Project for Statistical Computing	https://www.r-project.org/
Snapgene	Snapgene	https://www.snapgene.com:443/
Microsoft Office 365	Microsoft	https://www.office.com/
Prism version 8	GraphPad	N/A
BioRender App	BioRender	https://biorender.com

### Resource Availability

#### Lead Contact

Further information and requests for resources and reagents should be directed to and will be fulfilled by the Lead Contact, Sveta Chakrabarti (svetac@iisc.ac.in).

#### Materials Availability

*Drosophila* Prip-GFP line generated in this study is available from the Lead Contact without restriction.

#### Data and Code Availability

The accession number for the gene expression data reported in this paper is ArrayExpress: E-MTAB-8090.

### Experimental Model and Subject Details

#### *Drosophila* stocks and rearing under conventional and axenic conditions

*Canton*^*S*^ (*Can*^*S*^) and *w*^*1118*^ flies were used as wild-type controls. The following fly lines were used in this study: *w;UAS-upd2-IR* (NIG # 5988 R3), *w;UAS-upd3-IR* (Agaisse et al., 2003), *hml*Δ*Gal4,UAS-GFP* (Sinenko and Mathey-Prevot, 2004), *UAS-Duox-IR* and *UAS-IRC* (Ha et al., 2005), *UAS-Duox-IR-2* (BL33975, BDSC), *UAS-CatA* (BL24621, BDSC), *pxn-Gal4* (Stramer et al., 2005), *w;UAS-src42A-IR* (NIG # 7873, R3), *shark*^*1*^ (BL5865, BDSC), *w;UAS-drpr-IR* (BL36732, BDSC), *drpr*^*Δ5*^ (Freeman et al., 2003; backcrossed for 9 generations to remove lifespan effects obtained from Dr T. Kuraishi), *UAS-Prip-IR* (BL44464, BDSC), *UAS-Sod1* (BL24750, BDSC), *UAS-Sod2* (BL24494, BDSC), *w;UAS-Toll-IR* (BL31477, BDSC).

For RNAi (IR) studies, F1 progeny carrying one copy of the driver as well as one copy of the *UAS-IR* were raised at 18°C during their larval and pupal development, and then moved to 29°C for 8 days to activate the *UAS-IR* 3 days post-eclosion. *Drosophila* stocks were maintained using standard fly medium comprising of 8% cornmeal, 4% sucrose, 2% dextrose, 1.5% yeast extract, 0.8% agar, supplemented with 0.4% propionic acid, 0.06% orthophosphoric acid and 0.07% benzoic acid. All stocks were maintained at 25°C on a 12 h light/ 12 h dark-cycle unless otherwise stated.

Axenic *w*^*1118*^ flies were generated by bleaching embryos using 0.6% sodium hypochlorite. Briefly, embryos were washed in bleach for 3 mins twice, followed by a single 70% ethanol wash and final 3 washes with sterile MilliQ water. Finally, the embryos were transferred with the help of a sterile brush on autoclaved food. Sterile fly food was made by autoclaving at 121°C and 15 psi for 30 min and then immediately put on a horizontal shaker to prevent separation during cooling. Filter sterile preservatives were added under sterile conditions before the food was cooled. The presence of bacteria in gut homogenates was examined by PCR amplification of 16S rRNA genes using eubacterial primers (27F and 1492R), and by culturing the homogenates on mannitol agar or 1/10-strength tryptic soy agar.

### Method Details

#### Injury and infection experiments

For clean injury, flies were pricked in the thorax under the wing with a tungsten needle (Figure S1A). Female flies were used for experiments. For injury of axenic flies, the needle was sterilized by flaming and then a 15 min UV treatment. All injections of axenic flies were done under a laminar hood using ice to anaesthetize flies.

*Erwinia carotovora carotovora 15 (Ecc15)* is a Gram-negative bacterium described in (Basset et al., 2000) and *Enterococcus faecalis* (*E. faecalis*) is a Gram-positive bacterium. *Ecc15* was cultured overnight in Luria broth at grown at optimum growth conditions 29°C and *E. faecalis* was cultured overnight at 37°C. For septic injury experiments, *Drosophila* 3-4 days old adults were used. For septic injury, flies were pricked in the thorax with a needle dipped into a concentrated culture of *Ecc15* (OD_600_ ∼200) and shifted to 29°C for optimal bacterial proliferation and for *E. faecalis* (OD_600_ ∼2) the flies were maintained at 25°C. Flies in survival experiments were kept on medium without fresh yeast and survivors counted daily.

For all injury and infection experiments control flies were kept at the same time as the treatment and hemocytes harvested alongside the injured or infected flies.

#### Analysis of whole genome mRNA expression by RNA-seq

RNA-seq analysis was performed on 3 independent biological replicates. Hemocyte RNA from 60 adult *w*^*1118*^ of 3 to 5 days old whole females from was isolated by TRIzol extraction (Figure S1A) and purified with Direct-zol RNA kit (Zymo research). The quality of RNA was assessed using Agilent Bioanalyzer 2100 with a RNA 6000 nano kit. The RNA was checked for contamination of fat body tissue by preparing cDNA and carrying out RT-qPCR for the genes Fbp1 and Lsp2 (Figures S1G and S1H). Sequencing libraries were prepared using NEBNext Ultr II RNA Library Prep Kit for Illumina (NEB, E7770S) following and performed by Clevergene Biocorp Pvt Ltd. Bangalore, India. Briefly, 1 μg of total RNA was used as input for poly (A) mRNA enrichment using NEB Next Poly(A) mRNA Magnetic Isolation Module (NEB, E7490S), followed by fragmentation and reverse transcription to generate cDNA. Hairpin adaptor was ligated to fragmented double strand cDNA and USER enzyme was used to cleave the hairpin structure. Ampure beads were used to purify adaptor-ligated fragments and the purified product was amplified using NEBNext Multiplex Oligos for Illumina (NEB, #E7335S) to generate sequencing library. The library was quantitated using Qubit DNA High Sensitivity assay and library quality was checked with Bioanalyzer 2100 using Agilent 7500 DNA Kit. The libraries were sequenced using Illumina HiSeq 2500 to generate 2x150 bp reads. The sequencing data was processed in order to remove adaptor sequences and low-quality bases using Trim Galore (Babraham Bioinformatics). Sequencing data were checked using FastQC and MultiQC software, and all the samples passed the QC threshold (Q30 > 90%). The quality-trimmed data was mapped to the *Drosophila* genome (Release 6) using STAR v2 aligner. Gene expression was calculated as mapped read counts using FeatureCounts program. Differential expression analysis was performed using EdgeR after normalizing the expression counts based on trimmed mean of M values (TMM) method. GO clustering analysis was performed using FlyMine. RNaseq and statistical analysis was performed by Clevergene Biocorp Pvt Ltd. Bangalore, India. The FASTQ data files representing unique libraries are deposited in the ArrayExpress database (E-MTAB-8090).

#### qRT-PCR

For collecting hemocytes, 20 individuals were placed on a 30 μM filter of an empty Mobicol spin column (MOBITEC), then covered with glass beads and centrifuged for 20 minutes at 4°C, 10’000 rpm. This was done for twice for a total of 40 adult flies. The hemolymph was recovered and collected in 300 μL of Trizol (Takara Bio, 9108). Total RNA was extracted according to manufacturers’ instructions. Quality and quantity of RNA was determined using NanoDrop ND-1000 spectrophotometer (ThermoScientific). 1 μg of RNA was treated with 1U of DNase I (ThermoFischer Scientific, EN0521) following which cDNA was generated using Revertaid reverse transcriptase (ThermoFischer Scientific, EP0441). qRT-PCR was performed using dsDNA dye SYBR Green (Takara Bio, RR420A) on a Biorad CFX96 real time PCR machine. Expression values were normalized to *RpL32*.

#### Measurement and imaging of reactive oxygen species

*In vivo* measurement of ROS was done by injecting 100 nL of 20mM TCFB probe (boronate of 2-dicyanomethylene-3-cyano-4,5,5-trimethyl-2,5-dihydrofuran; kind gift from Prof H. Chakrapani, IISER Pune) into the thorax of 3-5-day old adult flies using a Sutter microinjector. The flies were allowed to recover for 30 mins and then imaged under an epifluorescence Axioplot imager (Zeiss). To visualize hemocytes transgenic *hml*Δ*Gal4,UAS-GFP* flies were injected with the TCFB2 probe (Sedgwick et al., 2017). Flies were imaged in three channels, i.e., GFP channel to visualize hemocytes, dsRed channel to visualize the TCFB probe, and brightfield to show the wounded cuticle. A merge of all these three channels is shown in Figures 2A–2D. For HEK293E cells, 20mM TCFB probe was preloaded on cells and then 100uM of H_2_O_2_ was added to different wells for imaging uptake.

For immunofluorescence, 3 to 5-day old females were dissected in 1X PBS, fixed for 20 minutes in PBS and 0.1% Tween 20 (PBT), and 4% paraformaldehyde. DNA was stained with 1/15000 dilution of 4’,6- diamidino-2-phenylindole DAPI (Sigma, D9542). The stained tissue was mounted in the antifading agent 2% n-propyl gallate (Sigma, P3130) in 80% PBS-glycerol.

#### Plasmids and Transgenic lines

Prip fosmid harboring *E. coli* strain was obtained from Source BioScience (United Kingdom). A line carrying the *Prip* transgene tagged with sGFP-V5-BLRP-FLAG and 15.4Kb region upstream and 5.8Kb downstream of the *Prip* gene, on the third chromosome was established. The fosmid clones FlyFos029594 (Source BioScience) was injected into the *y[1], w[^∗^], P{nos-phiC31int.NLS}X; PBac{y+-attP-3B}VK00033* (BL-32542). This stock has white eyes and no fluorescent eye markers, and the FlyFos lines were screened with the red fluorescent eye marker.

#### Cell Culture and Transfection

Human embryonic kidney (HEK) 293E cells were maintained DMEM/F-12 containing 120 mg/liter penicillin and 270 mg/liter streptomycin in the presence of 5% FBS (Invitrogen) at 37°C in a 5% CO2 humidified incubator. Transfections were performed with polyethyleneimine lipid. *Drosophila* Prip cDNA was obtained from DGRC (clone ID: LD27313) in the vector pOT-CG7777. The Prip gene was subcloned into the mammalian expression vector pcDNA2 under control of the CMV promotor using *EcoRV* and *XbaI*.

#### Imaging

The Prip FlyFos line was dissected and bled in 1XPBS containing 1mM phenylthiourea (PTU) on imaging dishes. These dishes were then imaged live using an Olympus FV 3000 confocal laser scanning microscope (CLSM) using a 60 × NA 1.4 oil objective with and without 10mM H_2_O_2_.

Subconfluent HEK293E cells were plated on 6 well dishes and transfected with pcDNA-EGFP and Prip. 48 h posttransfection, the wells were washed with DPBS, and incubated with 20mM TCFB probe in DPBS for 15 min at 37 °C. For HEK293E cell imaging was carried out on InCell Analyzer 6000 (GE Healthcare) Imaging system with 60x/0.7 N.A. Transfected cells were imaged with GFP and the TCFB probe with RFP using the 488 nm and 561 nm laser lines along with 525/20 and 605/52 nm bandpass emission filters respectively. After the addition of H_2_O_2_ uptake dynamics were obtained and time-lapse images were acquired 5 mins apart. Images were processed and quantifications were done on ImageJ.

For immunofluorescence, fly carcasses were dissected in 1X PBS, fixed for 20 minutes in PBS and 0.1% Tween 20 (PBT), and 4% paraformaldehyde; then stained with primary antibody [1/100 anti-dorsal (DSHB, 7A4)] in PBT + 2% BSA. Secondary staining was performed with Alexa488 anti-mouse antibodies (Invitrogen). The stained tissue was mounted in the antifading agent VECTASHIELD® with DAPI (Vector Labs.). The stained hemocytes were imaged with an Olympus FV 3000 confocal laser scanning microscope (CLSM) using a 60 × NA 1.4 oil objective and processed in ImageJ.

### Quantification and Statistical Analysis

Each experiment was repeated independently a minimum of three times, error bars represent the standard deviation of replicate experiments (unless otherwise indicated). All the experiments whether for survivals or qRT-PCR were repeated independently with 3 biological repeats (with 30 to 40 flies each). For normal data (qRT-PCR), one-way Analysis of Variance (ANOVA) was used to determine overall statistical difference and a Tukey’s test for Honest Significant Differences was used for multiple comparisons. Log rank tests were used for survival analysis. We have shown data from pooled experiments. All data was assumed as parametric and statistical significance was determined using Student’s t test, one-way ANOVA, two-way ANOVA or log–rank test on GraphPad Prism 8, and P values of < 0.05 = ^∗^, < 0.01 = ^∗∗^ and < 0.001 = ^∗∗∗^ were considered significant.

Figures 5A and 6 were created with BioRender (https://biorender.com).

## Supplemental Information

Document S1. Figures S1–S7 and Table S1

Document S2. Article plus Supplemental Information
