# Supplementary material for: Intramacrophage ROS Primes the Innate Immune System via JAK/STAT and Toll Activation
Source: Cell Rep. 2020 Nov 10;33(6):108368. doi: 10.1016/j.celrep.2020.108368 (PMC7662148; doi:10.1016/j.celrep.2020.108368)
Supplement: Document S1. Figures S1–S7 and Table S1 [file mmc1.pdf]

Cell Reports, Volume *33*

Supplemental Information

Intramacrophage ROS Primes the Innate Immune System via JAK/STAT and Toll Activation

Sveta Chakrabarti and Sandhya S. Visweswariah

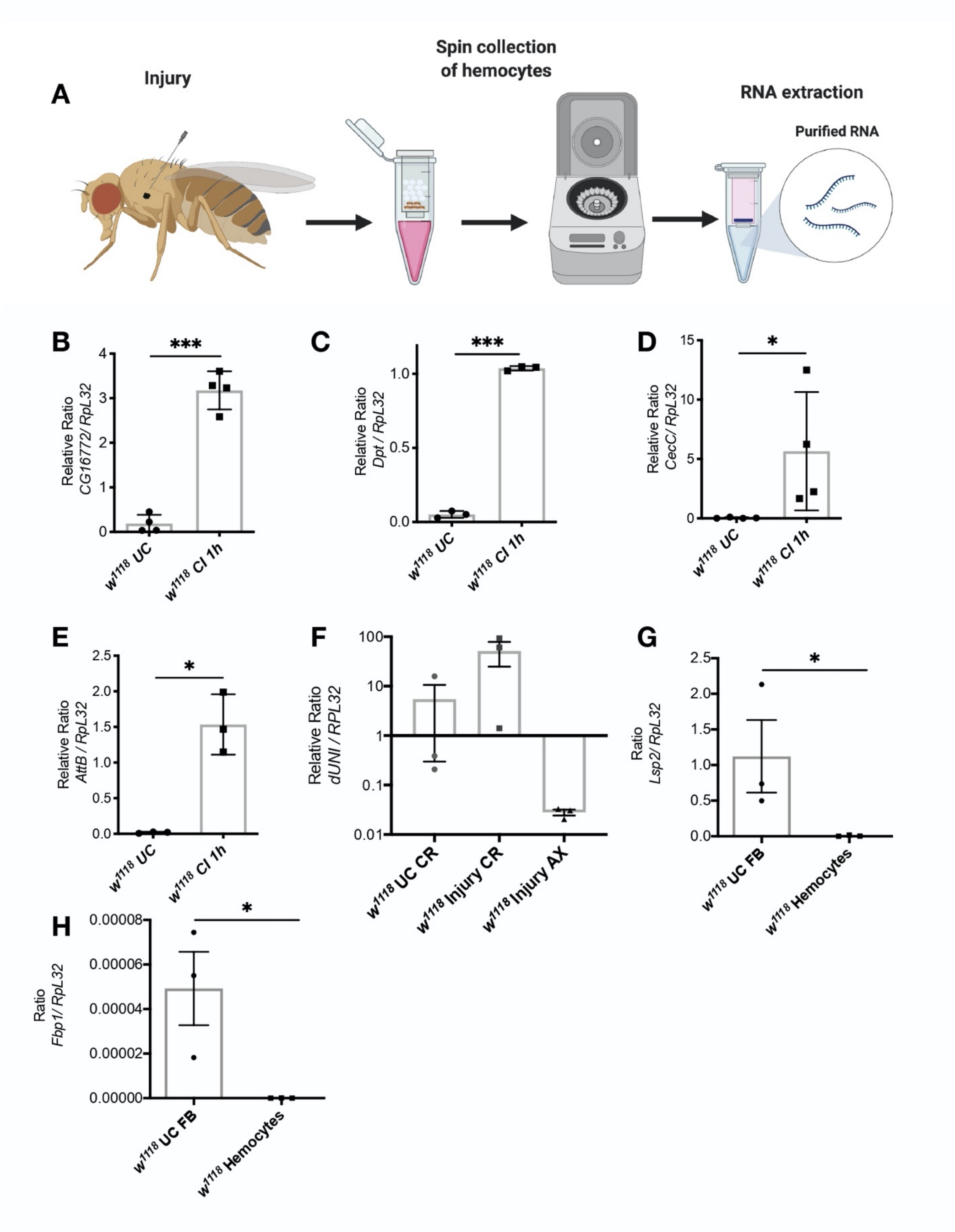

Fig S1. RNA-Seq of hemocytes after an injury. Related to Figure 1. (**A**) Experimental setup for collecting hemocytes from female adults after an injury. (**B-E**) The raw values for the most differentially expressed genes from the RNA-seq data in RT-qPCR experiments shown in wild-type (w^{1118}) hemocytes 1h following injury including (A) *CecC*, (B) *DptA*, (C) *AttB* and (D *CG16772*. ***: p< 0.0001, *: p< 0.05 and ns: non-significant as determined by one-way ANOVA, with post-hoc Tukey's test. Female flies were used for experiments in (A) and (B). (**F**) RT-qPCR experiments using a universal primer for bacteria. Axenic flies have no detectable bacteria as compared to the conventionally reared w^{1118} unchallenged (UC) and clean injury (CI) flies. (**G & H**) RT-qPCR in wild-type hemocytes collected by the spin column method shows little to no expression of *Fbp1* and *Lsp*.

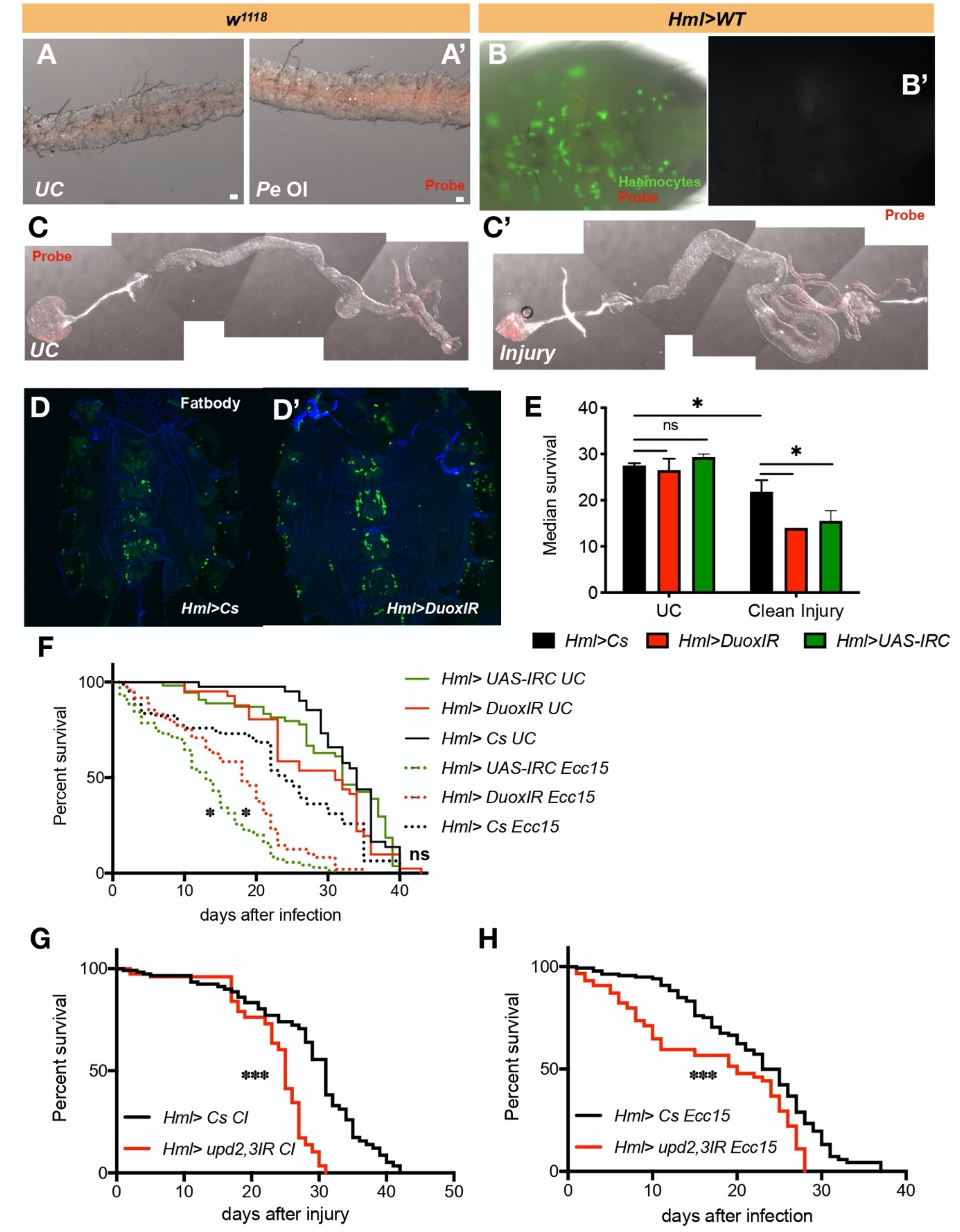

Fig S2. **Injury-induced ROS burst in hemocytes. Related to Figure 2.** (**A**) Hydrogen peroxide accumulates in the lumen of female intestines after oral infection (OI) with *Pseudomonas entomophila* pathogen validating the TCFB probe. (**B**) Representative images following feeding the TCFB probe (in red) before and after injury to the thorax using *hmlΔGAL4 > UAS-GFP* (green) female flies. There is little background red fluorescence of the TCFB probe in the intestine without injury, as well as systemically (abdomen imaged of entire fly). Flies were imaged in three channels, i.e GFP channel to visualize hemocytes, dsRed channel to visualize the TCFB probe, and brightfield to show the wounded cuticle. A merge of all these three channels is shown. (**C-C'**) Representative images of intestines from unchallenged and injured flies fed with the TCFB probe show no H_2O_2 accumulation in the midgut region of these intestines. The entire gut was imaged as sections, and a mosaic was constructed using

individual images with the help of FIJI software. (**D-D'**) The distribution of hemocytes in the fat body of wild-type and *Duox* RNAi flies using *hmlΔGAL4* > *UAS*-GFP. No difference in the abundance of hemocytes after the knockdown of *Duox* is seen. Merge image of DAPI channel and GFP channel is shown. (**E**) Two-way ANOVA, with a Bonferroni post-hoc test on the median survivals of the data shown in (Fig 2G). A significant difference in median survival between *hmlΔGAL4* > *UAS-DuoxIR* and *hmlΔGAL4* > *UAS-IRC* to the wild type control after injury is seen. (**F**) Flies with reduced ROS burst, i.e. *hmlΔGAL4* > *UAS-DuoxIR* and *hmlΔGAL4* > *UAS-IRC* show no difference in their longevity but an increased susceptibility after a septic injury. n = 60 flies per genotype pooled from three independent experiments. Log-rank test was used to determine statistical significance. n = 60 flies per genotype pooled from three independent experiments. Female flies were used for experiments, ***: p< 0.0001, **: p< 0.01, *: p< 0.05 and ns: non-significant. (**G-H**) Flies with reduced *upd2* and *upd3* expression by hemocytes, i.e. *hmlΔGAL4* > *UAS-upd2IR;UAS-upd3IR* showed higher susceptibility to septic injury with a clean injury (A) and *Ecc15* ((B). Log-rank test was used to determine statistical significance. n = 60 flies per genotype pooled from three independent experiments. Female flies were used for experiments, ***: p< 0.0001, **: p< 0.01, *: p< 0.05 and ns: non-significant.

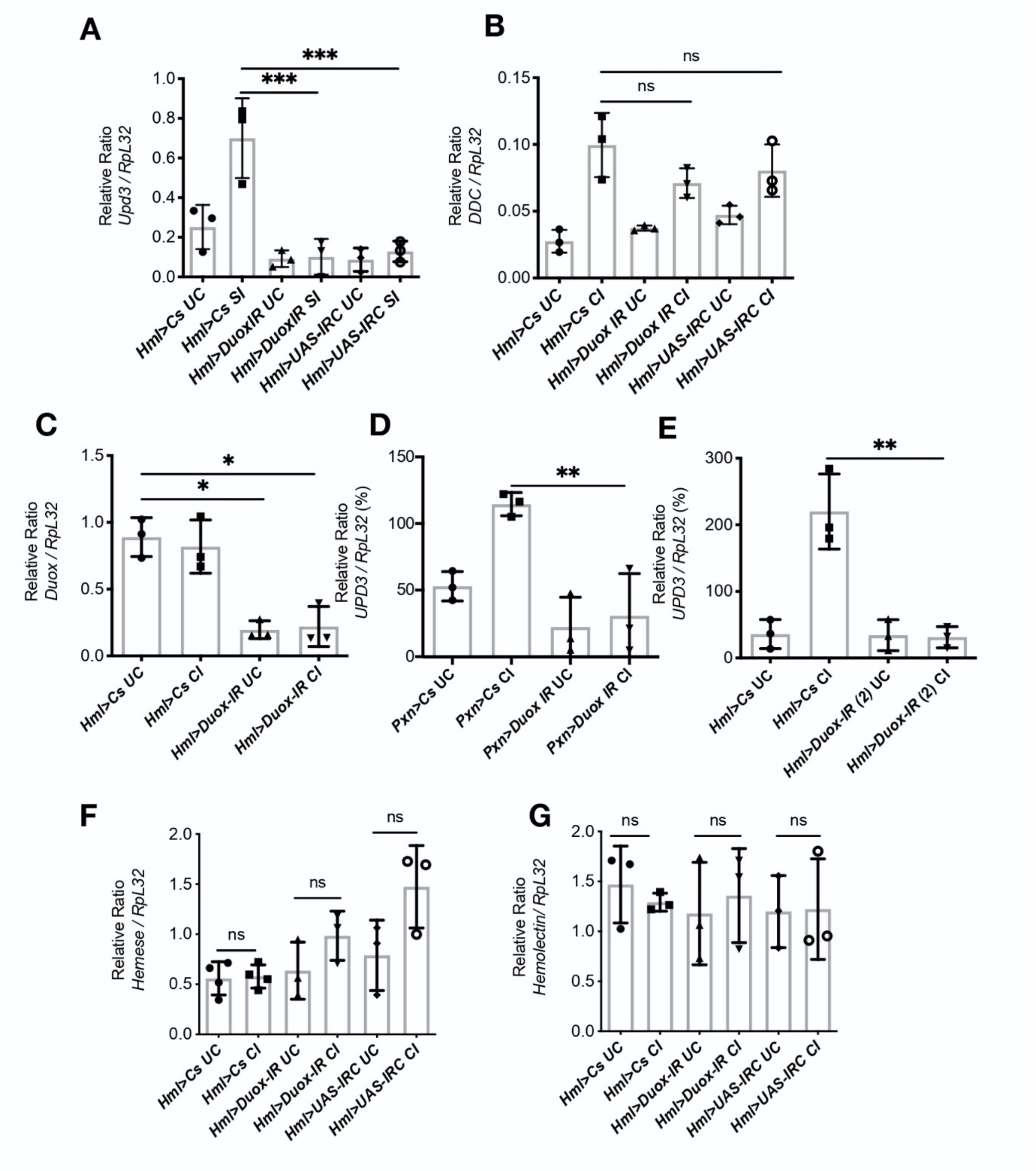

Fig S3. ***Duox* expression is essential for *upd3* expression after an injury by hemocytes. Related to Figure 2.** (**A-B**) *hemolectin* and *hemese* expression in wild-type flies and *hmlΔGAL4 > UAS-DuoxIR* and *hmlΔGAL4 > UAS-IRC* when normalized to *RpL32.* No change in hemocyte numbers as extrapolated through either *hemolectin* or *hemese* expression was observed in any genotype. (**C-D**) RT-qPCR in wild-type flies and *hmlΔGAL4 > UAS-DuoxIR* and *hmlΔGAL4 > UAS-IRC. ddc* expression remains unaffected on injury (C), while *upd3* expression is reduced upon septic injury (D). (**E**) *Duox* expression in wild-type and *hmlΔGAL4 > UAS-DuoxIR* flies, shows a robust knockdown of *Duox* in hemocytes. (**F-G**) Knockdown of both *Duox* in hemocytes using the *PxnGAL4* (F) driver and using an independent RNAi line for *Duox* with the *hmlΔGAL4* driver (G). RT qPCR analyses were performed with RNA prepared from hemocytes isolated from female adult flies. Mean values of at least three experiments (with 30 to 40 flies each) ± SD are shown. **: p< 0.01, *: p< 0.05 and ns: non-Significant as determined by one-way ANOVA, with post-hoc Tukey's test. UC: unchallenged; SI: Septic Injury for D & CI: clean injury for C;E-G.

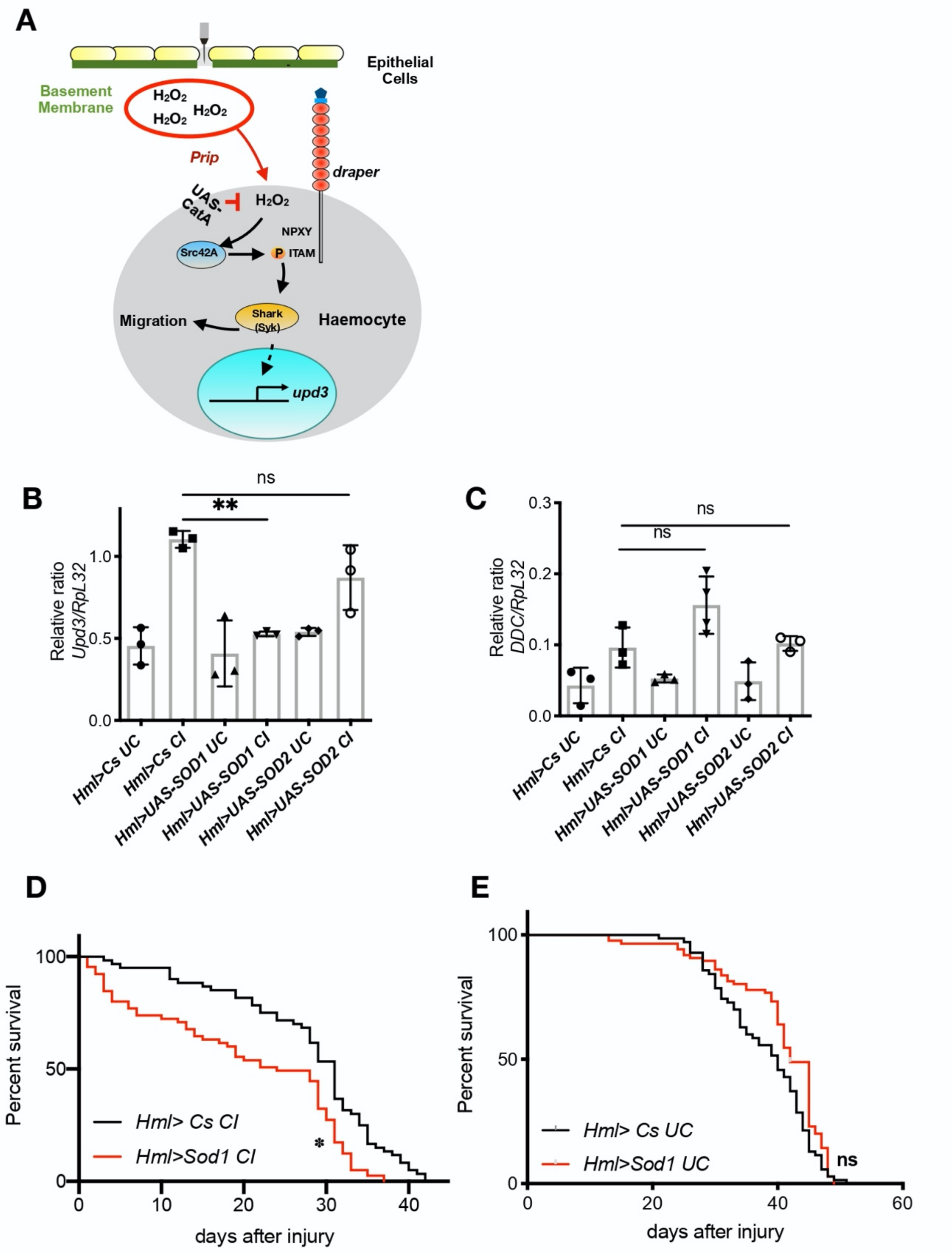

Fig S4. The accumulation of intracellular ROS in hemocytes is required for their activation and the survival of flies following an injury. Related to Figure 3. (**A**) Schematic illustrating how hemocyte migration depends on the kinase Src42A and its downstream target Shark and Draper upon wounding. UAS-Sod1 was used to reduced intracellular levels of ROS inside hemocytes. (**B & C**) RT-qPCR in wild-type and *hmlΔGAL4 > UAS-Sod1* and *hmlΔGAL4 > UAS-Sod2* flies. Overexpression of Sod1 (*hmlΔGAL4 > UAS-Sod1*) and Sod2 (*hmlΔGAL4 > UAS-Sod2*) lead to the reduction of ROS in the cytoplasm and mitochondria of hemocytes respectively. (**D**) Reduced ROS accumulation after an injury in *hmlΔGAL4 > UAS-Sod1* flies. Flies per genotype were pooled from three independent experiments and log-rank test used for comparing *hmlΔGAL4 > UAS-Sod1* (n = 59) and wild-type (*hmlΔGAL4 > Cs*, n = 60) adult flies. (**E**) Flies with reduced ROS accumulation in their cytoplasm after an injury (*hmlΔGAL4 > UAS-Sod1;* n = 61) showed no difference in their longevity as compared to the wild-type (*hmlΔGAL4 > CS*; n = 70). Log-rank test was used to determine statistical significance. n ≥ 60 flies per genotype pooled from three independent experiments.

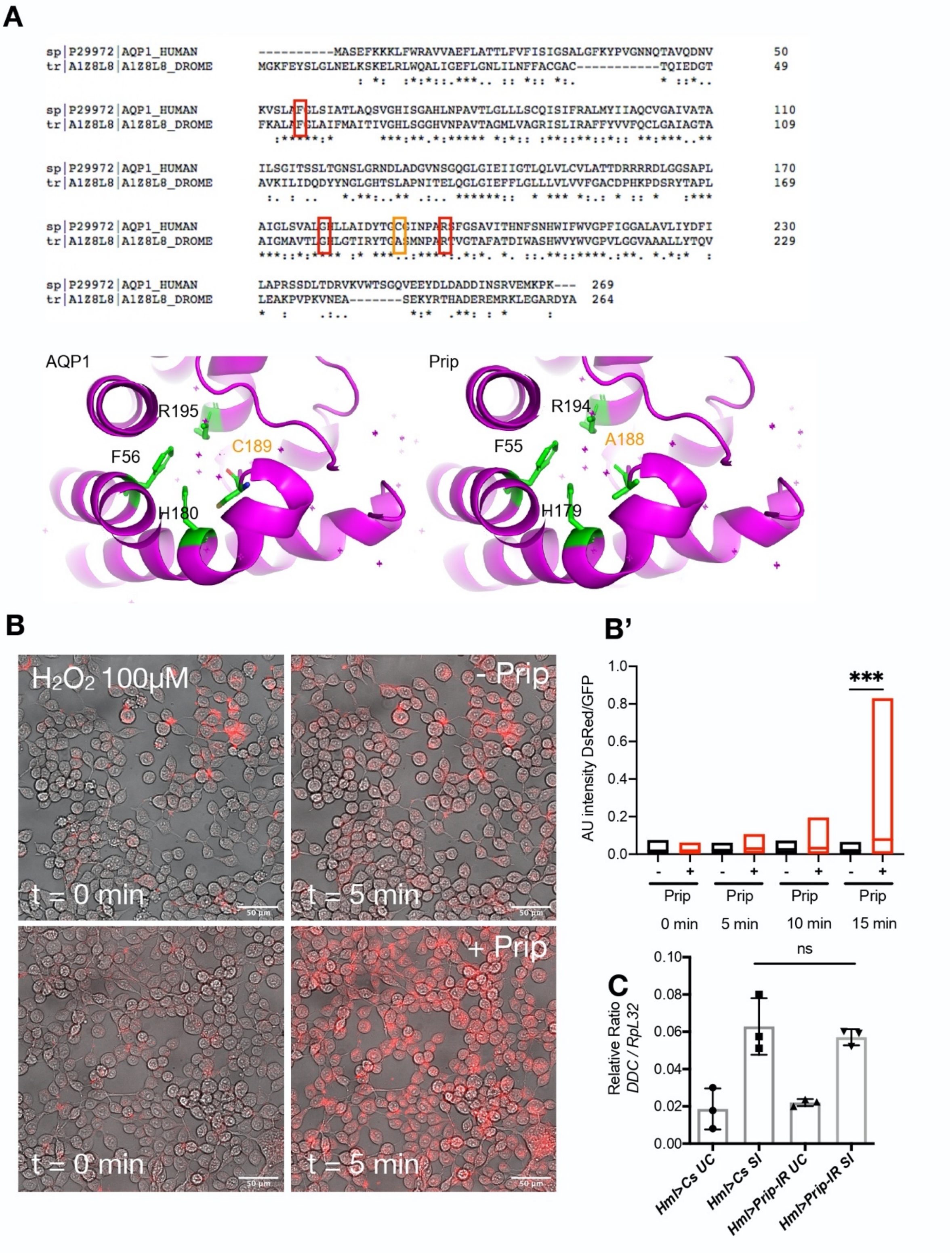

Fig S5. The accumulation of ROS is required for expression of *upd3* following injury. Related to Figure 3. (**A**) Alignment of *Drosophila* Prip protein with human aquaporin protein Aqp1 using Clustal Omega tool (Sievers et al., 2011). Amino acids critical channel function are highlighted in the sequence in red and orange color, and shown using a model of the two proteins. Aqp1 has a known structure (PDB: 1J4N). The residues have an arrangement that allows water and H_2O_2 to fit into the channel in Aqp1. The model of the fly Prip protein was calculated with the SWISS-MODEL program using the homologous structure found for AQP1 as a template (Waterhouse et al., 2018). Model showing the putative critical residues from the fly protein Prip using PyMOL Molecular Graphics System, version 2.0. (**B**) We expressed Prip CDS in HEK293 cells and used the TCFB probe to monitor kinetically the increase in fluorescence in cells where exogenous 100 uM H_2O_2 was added. Top panels are untransfected control cells, where 100uM H_2O_2 was added and increase in TCFB probe fluorescence was monitored over time.

Merge of brightfield and DsRed channel is shown. (**B'**) There was a significant increase in TCFB fluorescence over time in cells expressing Prip versus the vector control HEK cells at 100 uM exogenous H_2O_2 addition at the end of 15 mins imaging. The cells in all the panels were loaded with 5mM TCFB probe prior to the addition of H_2O_2. (**C**) RT-qPCR in wild-type flies and *hmlΔGAL4 > UAS-Prip-IR*. There is no difference in the expression of the wound-induced gene *ddc* after septic injury. **: $p < 0.01$, *: $p < 0.05$ and ns: non-Significant as determined by one-way ANOVA, with post-hoc Tukey's test.

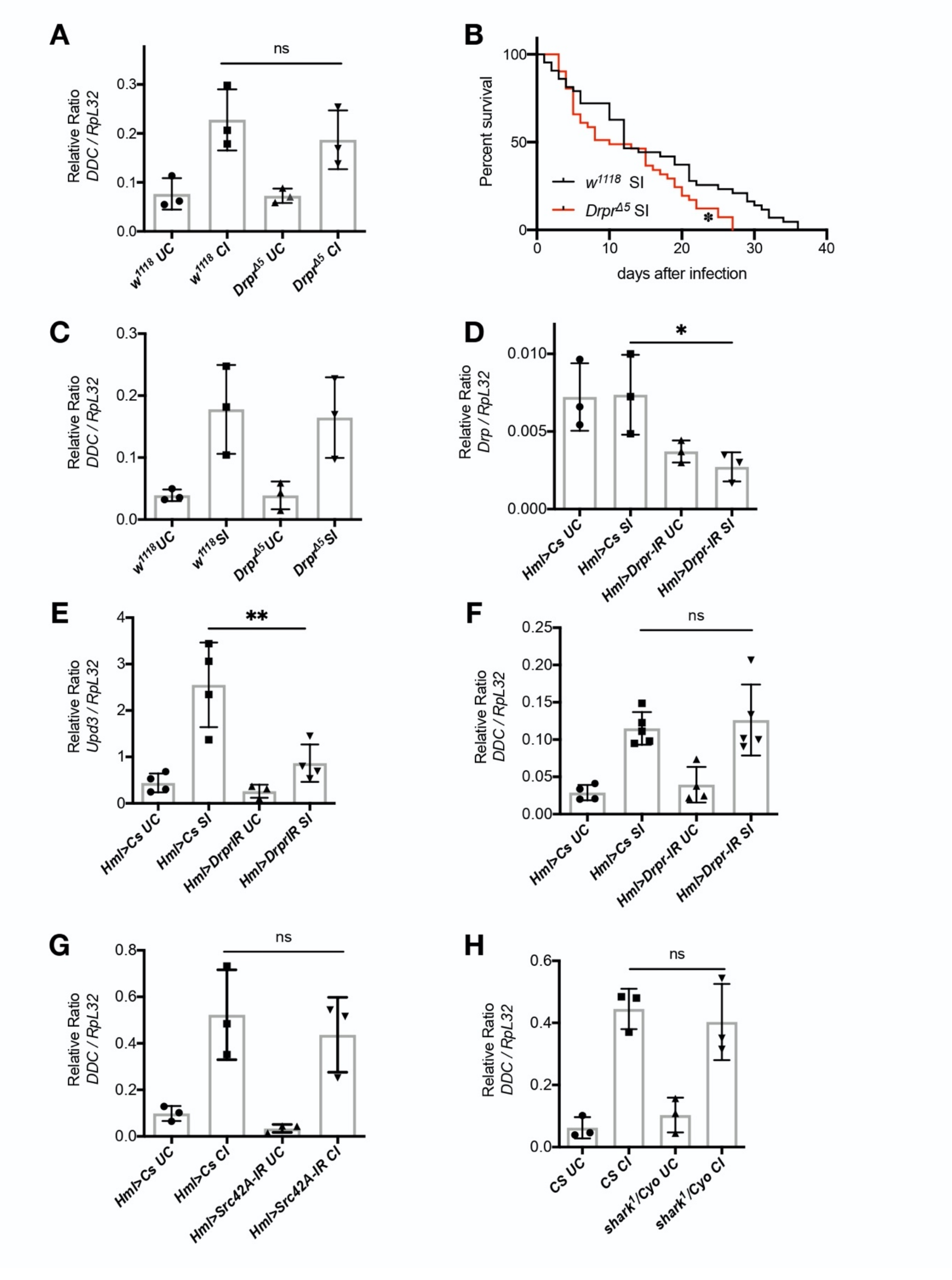

Fig S6. The Src42A/Shark/Draper pathway is required for expression of *upd3* following injury. Related to Figure 4. (**A**) *drpr*$^{\Delta5}$ (n = 146) mutant flies show no difference in their longevity as compared to the wild-type (n = 109). n ≥ 100 flies per genotype that is pooled from five independent experiments. Log-rank test used for comparing wild type (*w*^{1118}) and *drpr*$^{\Delta5}$ flies. (**B-C**) RT-qPCR of backcrossed *drpr*$^{\Delta5}$ flies showed no difference in the expression of *ddc* 1h after injury (B) and septic injury (C). (**D-F**) Expression of *drpr* with and without injury and *upd3* expression after injury in hemocytes as compared to their wild-type counterparts (*hmlΔGAL4* > *UAS-DrprIR* vs *hmlΔGAL4* > *CS*). (**G-H**) Hemocyte knockdown of *src42AIR* (*hmlΔGAL4* > *UAS-src42AIR*) and *shark*^{1} heterozygotes. No change in the expression of *ddc* expression 1h after injury is seen. For (**A**, **C-H**) ± SD are shown. ***: p< 0.0001. **: p< 0.01, *: p< 0.05. NS: non-significant, by one-way ANOVA, with post-hoc Tukey's test.

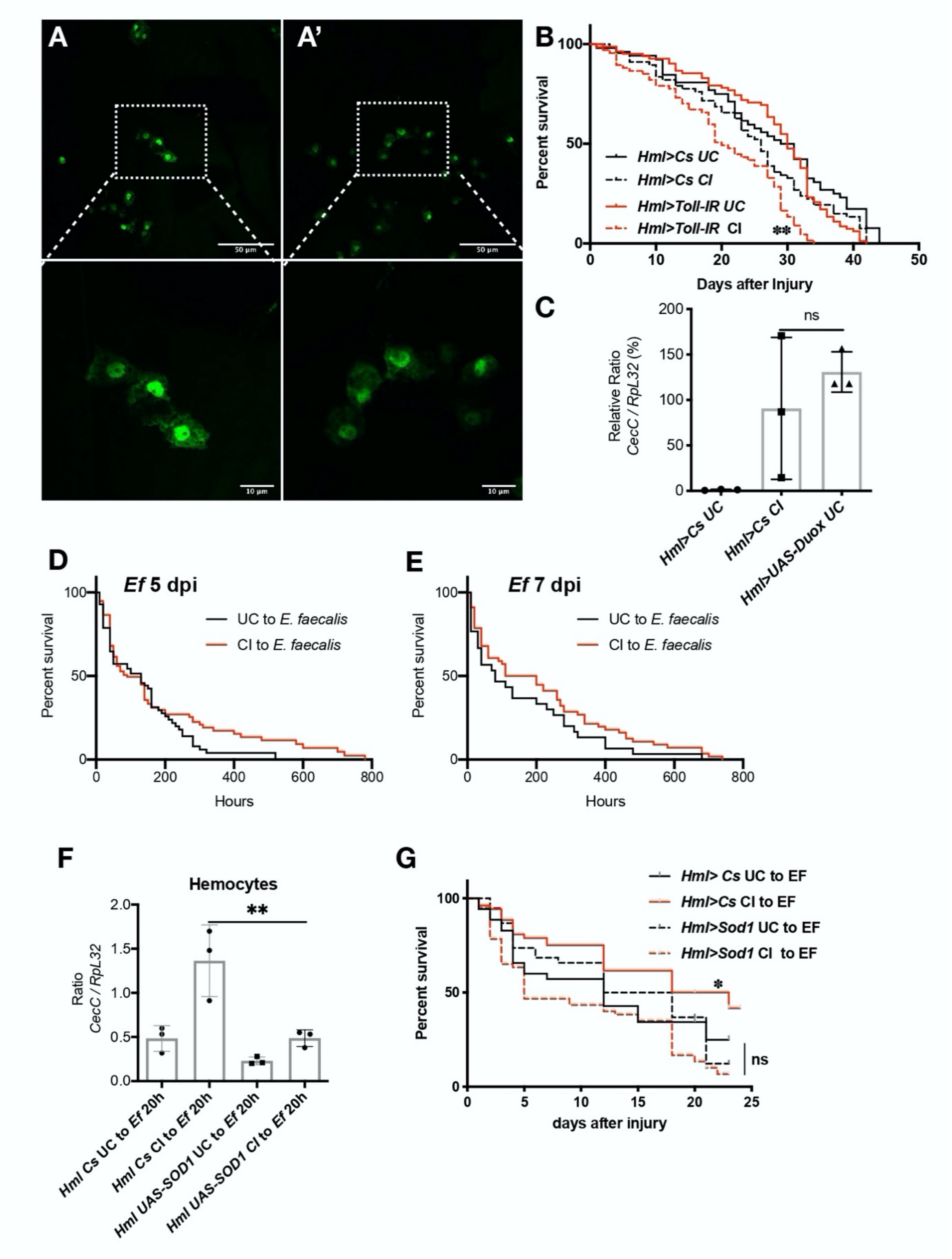

Fig S7. Toll signaling is active in hemocytes after wounding and lowering the ROS response following injury attenuates the protective effect on subsequent systemic infection. Related to Figure 5. (**A**) Immunostaining against dorsal of hemocytes of w^{1118} flies reveals that the dorsal transcription factor nuclear signal is activated upon clean injury of female flies. (**A'**) Nuclear signal is seen clearer in the white boxed panels below that are zooms of the panels above. (**B**) Flies with reduced Toll1 receptor after an injury, i.e. *hmlΔGAL4 > UAS-Toll1IR* show increased susceptibility to injury. A log-rank test was used to determine statistical significance. The injury was repeated independently 3 times on a minimum of 20 female flies per biological experiment. *hmlΔGAL4 > UAS-Toll1IR* (UC, n = 82; CI, n= 67) adult flies as compared to wild-type (*hmlΔGAL4 > Cs* UC, n = 52; CI, n= 67). P value < 0.001 between *hmlΔGAL4 > UAS-Toll1IR* CI and *hmlΔGAL4 > Cs* as determined by Log-rank test. (**C**) Ectopic

overexpression of *Duox* in adult fly hemocytes without injury stimulates the expression of *CecC* in hemocytes. (**D-E**) RT-qPCR of the AMPs *CecC* and *drosomycin* (*drs*) expression in the fat body of naïve vs injured flies w^{1118} flies that were subsequently infected with *E. faecalis* for 20h. There is little difference in the fat body response between naïve vs injured flies after infection with *E. faecalis*. UC: unchallenged; CI: clean injury; *Ef*: *E. faecalis*) ± SD are shown. *: $p< 0.05$ and ns: non-significant as determined by one-way ANOVA, with post-hoc Tukey's test. (**F**) RT-qPCR in wild-type and *hmlΔGAL4 > UAS-Sod1* flies in hemocytes of naïve vs injured flies that were subsequently infected with *E. faecalis* for 20h. Overexpression of Sod1 (*hmlΔGAL4 > UAS-Sod1*) that leads to the reduction of ROS in the cytoplasm was done by rearing the flies at 29°C for 3 days before the clean injury. The flies were subsequently shifted to 25°C at which temperature they were infected with *E. faecalis*. (**G**) Survival of flies following infection with *E. faecalis* as indicated in (Fig 5a). Flies per condition are pooled from at least three independent experiments. Log-rank test used for comparing wild type and *hmlΔGAL4 > UAS-Sod1* (*hmlΔGAL4 > Cs*; UC, n = 30 & CI, n = 30; *hmlΔGAL4 > UAS-Sod1*; UC, n = 30 & CI, n = 55).

Table S1. Quantitative PCR primers. Related to STAR Methods.

Oligonucleotides		
RpL32 Forward: GACGCTTCAAGGGACAGTATCTG	This paper	N/A
RpL32 Reverse: AAACGCG-GTTCTGCATGAG	This paper	N/A
AttB Forward: GCAATGGAGCTGGTCTGGAT	This paper	N/A
AttB Reverse: CCGATTCCTGGGAAGTTGCT	This paper	N/A
CG16772 Forward: ACCGATGATACGCATCTGCC	This paper	N/A
CG16772 Reverse: CGTGGTGGTTTCTGGCTTTG	This paper	N/A
CecC Forward: GCATTGGACAATCGGAAGCC	This paper	N/A
CecC Reverse: TTGCGCAATTCCCAGTCCTT	This paper	N/A
CG11892 Forward: ATGTCGGTTTCGGATCTGATTG	This paper	N/A
CG11892 Reverse: TTTCTTCCGGCACAAGAGACT	This paper	N/A
upd-3 Forward: GCGGGGAGGATGTACC	This paper	N/A
upd-3 Reverse: GTCTTCATGGAATGAGCC	This paper	N/A
ddc Forward: GAACGGAAGTAAAGCTCGGCAACAAG	This paper	N/A
ddc Reverse: CCTTGAACTCCGGCGCCTCC	This paper	N/A
Hemese Forward: GTTTTCCTGGCACTGTTCACT	This paper	N/A
Hemese Reverse: GGACGGTTGCTATGTATTGGTT	This paper	N/A
Hemolectin Forward: GGTTATGGCGGGATAAAGACG	This paper	N/A
Hemolectin Reverse: GTTGCCCTGACTTCCCTGG	This paper	N/A
dUNI Forward: AGAGTTTGATCCTGGCTCAG	This paper	N/A
dUNI Reverse: CTGCTGCCTTCCGTA	This paper	N/A
Fbp1 Forward: CTTCGCCGTAATGTGGTCTAC	This paper	N/A
Fbp1 Reverse: GAGCTTGAGTGTCCTCACGA	This paper	N/A
Lsp2 Forward: CTTCCAGCACGTCGTCTACTG	This paper	N/A
Lsp2 Reverse: CCCTGCATATCATCACGGAACA	This paper	N/A
